# Enhanced Vaccine Immunogenicity Enabled by Targeted Cytosolic Delivery of Tumor Antigens into Dendritic Cells

**DOI:** 10.1021/acscentsci.3c00625

**Published:** 2023-09-14

**Authors:** Nicholas L. Truex, Aurélie Rondon, Simon L. Rössler, Cameron C. Hanna, Yehlin Cho, Bin-You Wang, Coralie M. Backlund, Emi A. Lutz, Darrell J. Irvine, Bradley L. Pentelute

**Affiliations:** † Department of Chemistry, Massachusetts Institute of Technology, 77 Massachusetts Avenue, Cambridge, Massachusetts 02139, United States; ‡ Department of Chemistry and Biochemistry, University of South Carolina, 631 Sumter Street, Columbia, South Carolina 29208, United States; § Department of Materials Science and Engineering, Massachusetts Institute of Technology, 77 Massachusetts Avenue, Cambridge, Massachusetts 02139, United States; ∥ The Koch Institute for Integrative Cancer Research, Massachusetts Institute of Technology, 500 Main Street, Cambridge, Massachusetts 02139, United States; ⊥ Department of Biological Engineering, Massachusetts Institute of Technology, 77 Massachusetts Avenue, Cambridge, Massachusetts 02139, United States; # Ragon Institute of Massachusetts General Hospital, Massachusetts Institute of Technology and Harvard University, 400 Technology Square, Cambridge, Massachusetts 02139, United States; ¶ Howard Hughes Medical Institute, 4000 Jones Bridge Road, Chevy Chase, Maryland 20815, United States; □ Center for Environmental Health Sciences, Massachusetts Institute of Technology, 77 Massachusetts Avenue, Cambridge, Massachusetts 02139, United States; ● Broad Institute of MIT and Harvard, 415 Main Street, Cambridge, Massachusetts 02142, United States

## Abstract

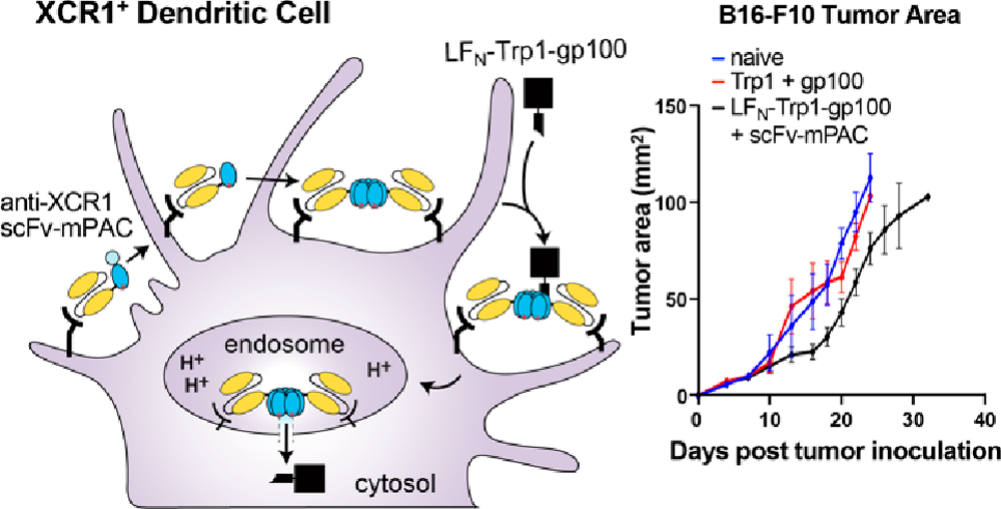

Molecular vaccines comprising antigen peptides and inflammatory cues make up a class of therapeutics that promote immunity against cancer and pathogenic diseases but often exhibit limited efficacy. Here, we engineered an antigen peptide delivery system to enhance vaccine efficacy by targeting dendritic cells and mediating cytosolic delivery. The delivery system consists of the nontoxic anthrax protein, protective antigen (PA), and a single-chain variable fragment (scFv) that recognizes the XCR1 receptor on dendritic cells (DCs). Combining these proteins enabled selective delivery of the N-terminus of lethal factor (LF_N_) into XCR1-positive cross-presenting DCs. Incorporating immunogenic epitope sequences into LF_N_ showed selective protein translocation in vitro and enhanced the priming of antigen-specific T cells in vivo. Administering DC-targeted constructs with tumor antigens (Trp1/gp100) into mice bearing aggressive B16–F10 melanomas improved mouse outcomes when compared to free antigen, including suppressed tumor growth up to 58% at 16 days post tumor induction (*P* < 0.0001) and increased survival (*P* = 0.03). These studies demonstrate that harnessing DC-targeting anthrax proteins for cytosolic antigen delivery significantly enhances the immunogenicity and antitumor efficacy of cancer vaccines.

## Abstract 2

A cancer vaccine targeting the XCR1 receptor on dendritic cells uses two engineered anthrax proteins, protective antigen (mPAC) and lethal factor (LF_N_), and a single-chain variable fragment (scFv).

## Introduction

Immunotherapies have gained significant traction over the past two decades for the treatment of cancer and infectious diseases.^1^ Cancer vaccines are a particularly interesting class of immunotherapy, with the potential to provide either prophylactic or therapeutic effects through the stimulation of a patient’s adaptive immune system.^2^ After considerable efforts to develop anticancer immunizations, one therapeutic cancer vaccine is now FDA approved for treating refractory prostate cancer (Sipuleucel-T, Provenge). The advent of new cancer vaccine platforms, particularly when combined with immune-checkpoint blockade (ICB), not only offer the promise of treating additional cancer types but also provide long-term remission through immune-memory responses.^3^ Nonetheless, successful elicitation of potent antitumor T cell responses after vaccination in humans, especially cytotoxic T cell responses, has thus far been challenging.^4^ Vaccine platforms comprising proteins, peptides, nucleic acids (DNA or RNA), viral vectors, or immune cells offer new avenues to overcome these challenges and to provide more effective vaccines against cancer.^2,5,6^

Delivering target antigens into specific cell targets has emerged as a promising way to boost vaccine immunogenicity. Antigen presenting cells (APCs) provide ideal targets because these cells specialize in proteolytic processing and loading of antigens onto the peptide-binding groove of human leukocyte antigen (HLA) molecules (or major histocompatibility complex (MHC) molecules) for priming of T cells. Populations of APCs include dendritic cells (DCs), macrophages, and B cells, but DCs that participate in cross-presentation are considered the most efficient APCs for priming cytotoxic and immune-memory T cell responses.^7−11^ Developing immunotherapies that target cross-presenting DCs is challenging because closely related DC populations can exhibit opposing activity.^12^ For example, CD8^+^ DCs exhibit antigen cross-presentation and favor cytotoxic T lymphocyte (CTL) responses, while CD8^–^ DCs favor non-CTL responses.^13,14^

Conventional type 1 dendritic cells (cDC1) efficient at antigen cross presentation are further identified by several unique receptors, including DEC-205, XCR1, and Clec9A.^15^ In particular, expression of the chemokine XCR1 receptor was observed in CD8^+^ DCs, but not in T cells, B cells, NK cells, or plasmacytoid DCs (pDCs).^16^ cDC1s are located in nonlymphoid tissues and in the marginal zone of the spleen and possess the ability to migrate in lymph nodes. While cDC2s activate CD4^+^ T cells, the cDC1 subset is the most effective for CD8^+^ T cell priming and for driving cell-mediated response via the cross presentation of exogenous and endogenous antigens to T cells.^17,18^ In cancer patients, the presence of cDC1s in the tumor microenvironment was correlated to a better survival and a higher response to anti-PD1 checkpoint blockade.^19,20^ Therefore, cDC1s are considered to play a critical role in antitumor immunity and represent a particularly attractive target for the development of cancer vaccines. Only one ligand is known to bind the XCR1 receptor of cDC1s, which is the chemokine XCL1 that induces CD8^+^ DC migration and maturation.^16^ Previous efforts to develop vaccines based on targeting XCR1^+^ DCs include fusing antigens to XCL1 or a monoclonal XCR1-specific IgG, which have been shown to enhance priming of CTL responses.^21−23^ Nonetheless, simply targeting the XCR1 receptor does not ensure uptake by intracellular compartments, which limits loading onto class I MHC molecules and reduces cross-presentation to T cells.^24,25^

Engineered bacterial toxins are an emergent delivery platform for shuttling therapeutic proteins into mammalian cells^26^ and may offer an effective approach to maximize the cytosolic delivery of vaccine antigens.^27,28^ In particular, the two nontoxic components of the anthrax delivery system, protective antigen (PA) and the N-terminus of lethal factor (LF_N_), have been shown to efficiently transport non-native cargo into the cytosol of cells, including more than 30 different peptides, proteins, and even small molecules.^29^ Delivery through binding of the native anthrax receptor is readily achieved by coadministering PA and LF_N_ fused to the desired cargo. Changing the receptor specificity of PA can enable targeted, PA-mediated delivery into specific cell types.^30−34^ Achieving targeted delivery with the PA/LF_N_ system through non-native receptors requires development of fusion proteins with PA and a receptor-targeting protein while maintaining the function of both components. Developing a receptor-targeting PA fusion protein is a work-intensive process that has typically proved challenging. As a result, only a few receptor-targeting PA variants have been developed to date.

We recently introduced a generalizable workflow for incorporating receptor-targeting proteins into PA with a defined chemical bond.^35^ Essential to this workflow is a triple mutant PA, called mPAC, which contains two mutations that ablate binding to native anthrax receptors and a third mutation that provides a single cysteine residue for bioconjugation. Bioconjugation has allowed facile incorporation of mPAC onto targeting proteins, including antibodies, from diverse expression systems without hindering the preparation and native function of the conjugated components. The bioconjugation workflow has accelerated the development of new receptor-targeting PA variants in our laboratory and is enabling in-depth preclinical studies on their therapeutic efficacy. Over time, we anticipate that the PA conjugates will be further developed as fusion proteins or used directly as conjugates in clinical settings.

**Figure 1:**
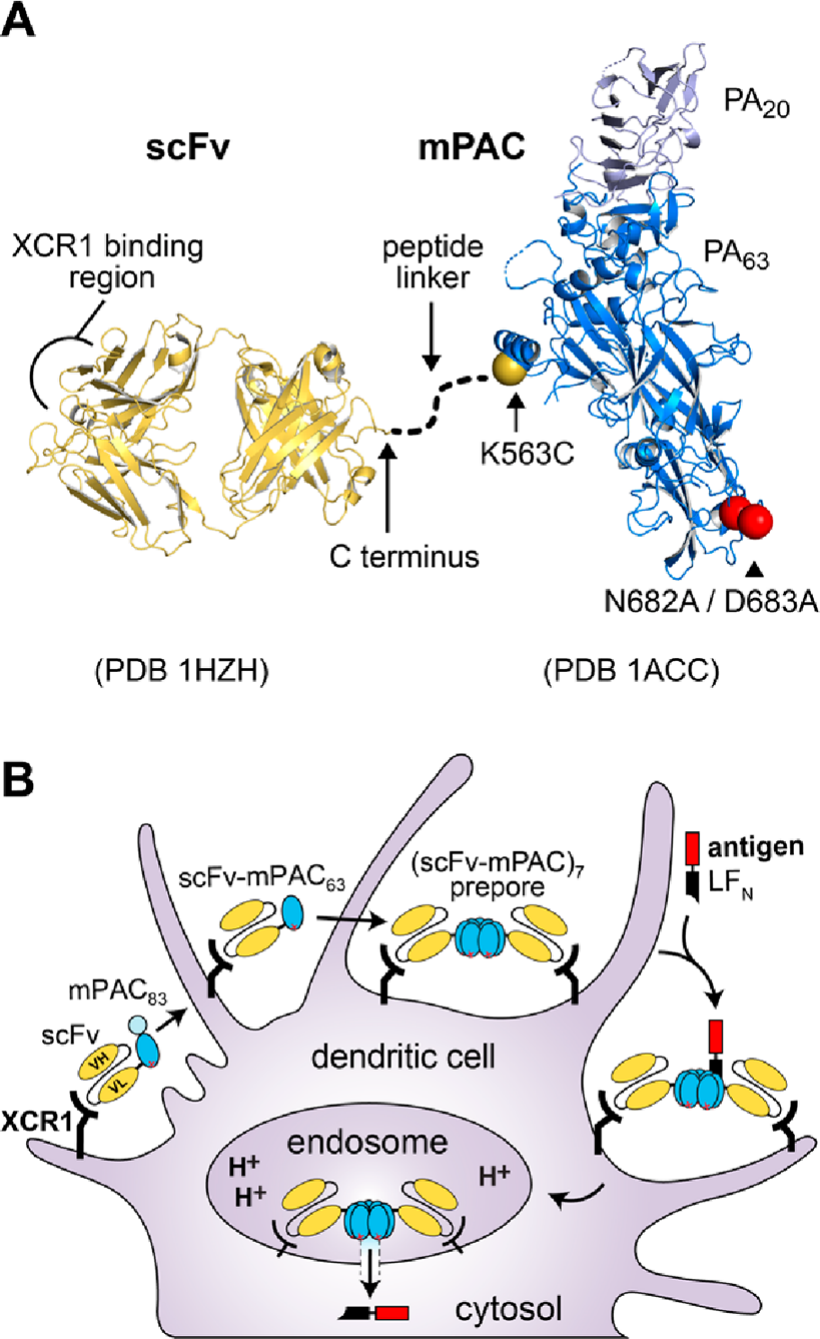
Engineered anthrax proteins for targeted vaccine delivery into cross-presenting dendritic cells. (A) Designs of the engineered components: triple mutant protective antigen (mPAC), which enables side-chain bioconjugation and ablates binding to native anthrax receptors; single-chain variable fragment (scFv), which recognizes the XCR1 receptor; and a linker peptide (dotted line), which connects the scFv and mPAC. (B) Envisioned mechanism of translocation for scFv-mPAC (scFv-mPAC_83_), which exhibits recognition of the XCR1 receptor, proteolytic cleavage (scFv-mPAC_63_), oligomerization (scFv-mPAC)_7_, and cytosolic delivery of the N-terminus of lethal factor (LF_N_) with an appended antigen peptide (red).

Here, we combined mPAC with a single-chain variable fragment (scFv) that recognizes the XCR1 receptor (Figure 1A).^14^ A peptide linker connects the two domains, enabling protein translocation with full-length mPAC (mPAC_83_). The resulting scFv-mPAC (scFv-mPAC_83_) translocates antigenic cargo fused with LF_N_, in which the scFv targets XCR1-positive cells and the mPAC exerts the conventional PA-mediated translocation mechanism (Figure 1B). This mechanism includes (1) scFv-mPAC_83_ binding to the XCR1 receptor; (2) proteolytic cleavage of scFv-mPAC_83_ into two components, scFv-mPAC_63_ and mPAC_20_; (3) scFv-mPAC_63_ oligomerization into a heptameric prepore; (4) binding of three or four LF_N_ molecules to the scFv-mPAC_63_ prepore; (5) endocytosis of the prepore complex, followed by the formation of an active transmembrane pore upon acidification of the endosome; and (6) PA-mediated translocation of the LF_N_ molecules into the cytosol. We anticipated that targeting XCR1 with the scFv would mimic functions of the native XCL1, by supporting induction of CD8^+^ DC migration and maturation.^16^ Moreover, we anticipated that facilitating cytosolic antigen delivery into XCR1^+^ DCs would enhance the potency of the antigen cargo by providing access to the class I MHC antigen loading pathway.^24,25^ Also, we envisioned that this approach would limit off-target delivery into other cell types, requiring lower antigen amounts to achieve effective CTL responses.

Our studies show that anti-XCR1 scFv-mPAC selectively targets and delivers protein cargo into XCR1-positive cells. In vitro studies demonstrate that the translocation mechanism operates in a receptor-dependent and PA-mediated fashion. In vivo biodistribution studies highlight that scFv-mPAC accumulates in the lymph nodes and spleen and is taken up by antigen-presenting cells. In vivo vaccination studies show that antigen delivery utilizing LF_N_/scFv-mPAC enhances antigen-specific immunogenicity, inhibits tumor growth in tumor-bearing mice, and improves survival. These studies show promise for further therapeutic development, particularly for cancer immunotherapy.

## Results

### Design and Preparation of a DC-Targeting Single-Chain Variable Fragment (scFv)

**Figure 2:**
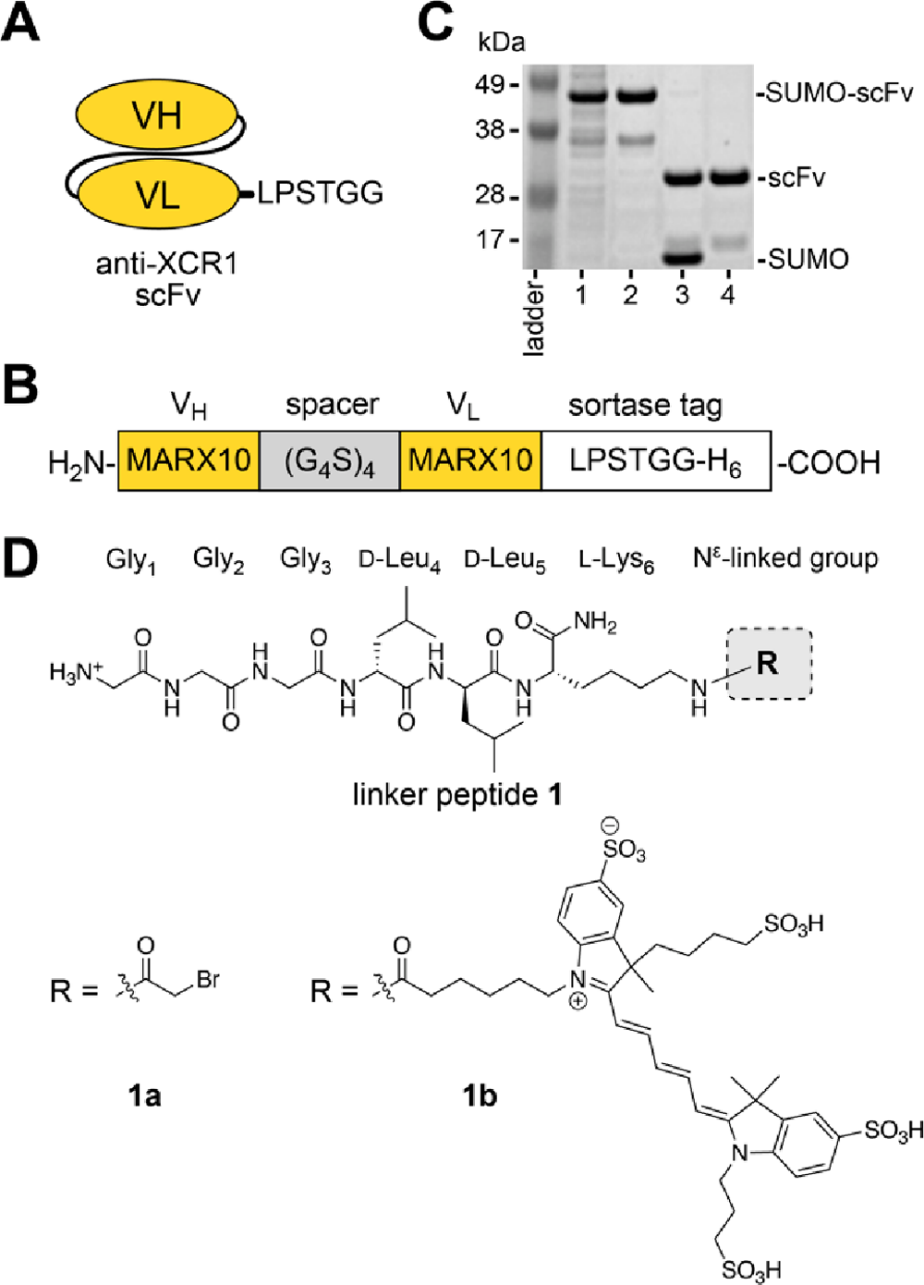
Development of an anti-XCR1 scFv. (A, B) Design of the anti-XCR1 scFv, which encodes variable heavy (V_H_) and light (V_L_) chains from a parent anti-XCR1 IgG (MARX10). The scFv also contains a hydrophilic region (G_4_S)_4_ between the V_H_ and V_L_ chains and a sortase recognition tag (LPSTG_2_H_6_) that enables protein ligation. (C) Coomassie-visualized SDS-PAGE gel of the recombinant scFv throughout the bacterial expression and purification steps: (1) whole cell lysate; (2) Ni NTA purification; (3) treatment with SUMO protease; and (4) ion-exchange (HiTrap Q HP) chromatography. (D) Linker peptide **1**, which included **1a** (R = α-bromoacetyl group) and **1b** (R = Alexa Fluor 647 (AF647)).

Previously, we introduced a targeted protein delivery platform that contains anthrax protective antigen (PA_83_) combined with a receptor-binding protein, including a full-length antibody or an scFv.^34,35^ These constructs selectively target cancer cells and translocate toxic payloads fused to the N-terminus of lethal factor (LF_N_).^34,35^ Here, we aimed to engineer the anthrax proteins as a nontoxic delivery platform for cancer immunotherapy, enabled by targeting DCs and facilitating cytosolic delivery of antigen peptides. We developed a recombinant scFv fragment that recognizes the XCR1 receptor of DCs, which encodes the variable heavy (V_H_) and light (V_L_) chains from a parent anti-XCR1 monoclonal antibody (MARX10) (Figure 2A,B).^14,36^ We incorporated a (G_4_S)_4_ spacer sequence between the V_H_ and V_L_ chains and a sortase-recognition tag to enable enzymatic ligation at the C-terminus (Figure 2B,C; Table S1). The scFv was prepared using recombinant expression in *E. coli*, followed by purification (Figure 2C) and characterization by LC-MS (Figure S1). We also synthesized two peptides for the ligation called linker peptides **1** (Figure 2D). The peptides each contained three Gly residues for sortase ligation and two d-Leu residues to impart proteolytic stability. We varied the peptides at the N^ε^-lysine position to contain an acetyl bromide (peptide **1a**) and an AlexaFluor-647 (AF647) fluorophore (peptide **1b**), followed by purification using RP-HPLC and characterization by LC-MS (Figure S2).

### Conjugating scFv to Protective Antigen

**Figure 3:**
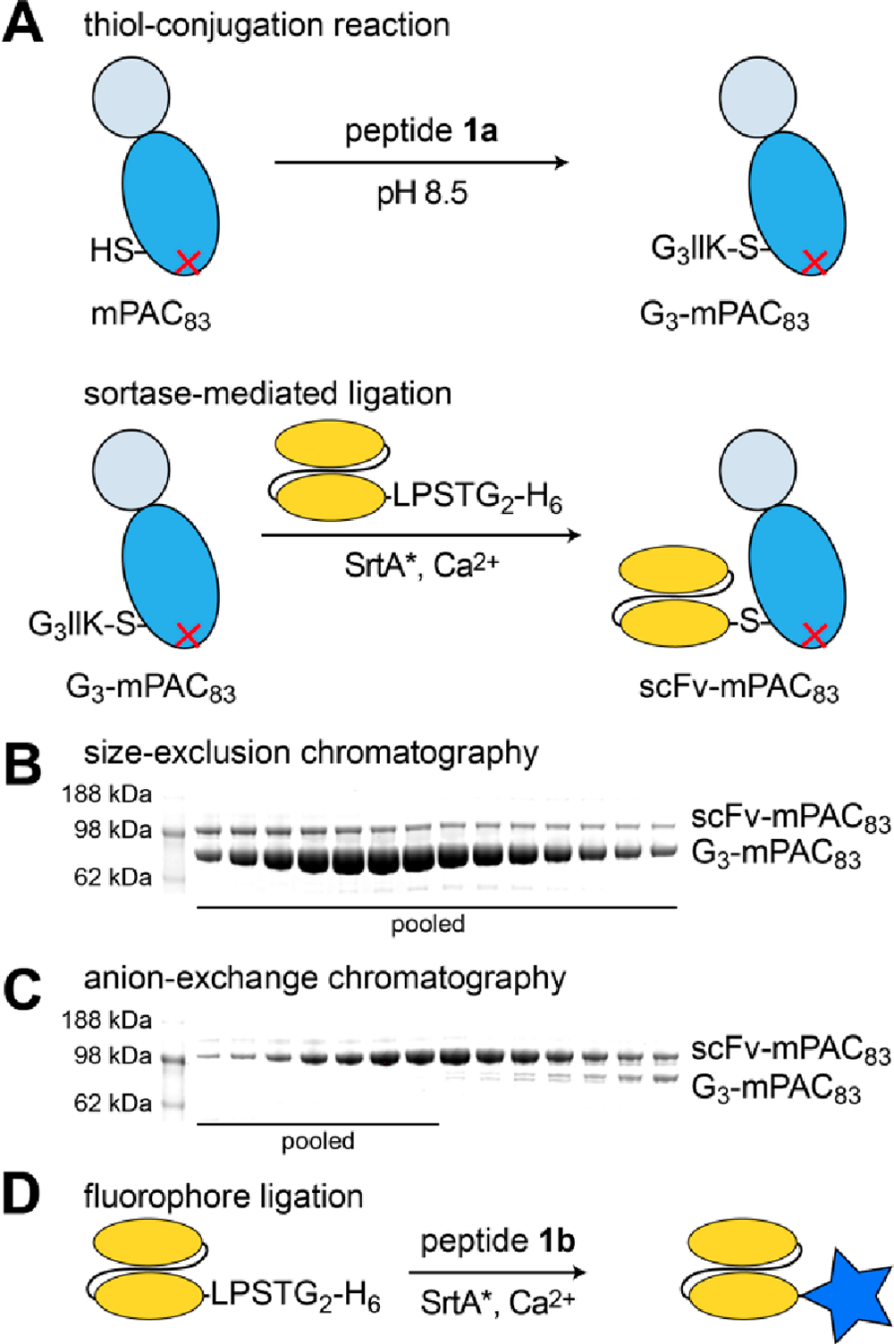
Engineering a DC-targeting anthrax protective antigen. (A) Schematics for the protein ligation of mPAC_83_ and an anti-XCR1 scFv: thiol-conjugation reaction with peptide **1a**, followed by sortase-mediated ligation with the scFv (scFv-LPSTG_2_-H_6_). (B, C) Coomassie-visualized SDS-PAGE gels of fractions obtained from size-exclusion (HiLoad 16/600 Superdex 200) and anion-exchange (HiTrap Q HP) chromatography. (D) Schematic of the sortase-mediated ligation reaction for scFv and peptide **1b**.

We conjugated the scFv to two previously reported PA mutants: (1) mPAC, which is a triple mutant PA[N682A, D683A, K563C] that permits bioconjugation but does not bind to native anthrax receptors; and (2) mPAC[F427A], which is a translocation-deficient homologue of mPAC that provides a negative control.^35,37,38^ The mPAC and mPAC[F427A] were produced through recombinant expression in *E. coli*, followed by purification using anion-exchange (AEX) chromatography and characterization by LC-MS (Figure S3).^39^ Each protein was combined with anti-XCR1 scFv by a two-step bioconjugation procedure. Figure 3A illustrates the procedure for mPAC (mPAC[F427A] is not shown). First, mPAC was incubated with peptide **1a** (60 min, pH 8.5) to conjugate the peptide onto the Cys_563_ residue, which gave G_3_-mPAC (Figure S4). Second, sortase-mediated ligation with the anti-XCR1 scFv-LPSTG_2_-H_6_ afforded the corresponding scFv-mPAC construct (Figure S5).^35^ SDS-PAGE analysis showed purified fractions of scFv-mPAC after size-exclusion chromatography (SEC), followed by AEX chromatography (Figure 3B,C). The scFv-mPAC[F427A] was prepared in a similar fashion, which provided a translocation-deficient homologue as a negative control. We also prepared a AF647-labeled scFv to enable cell-binding studies. We used sortase-mediated ligation to combine scFv with peptide **1b**, which was purified by SEC (Figure 3D). Successful preparation of the scFv-AF647 construct was confirmed by LC-MS analysis (Figure S6).

### Selective Protein Delivery into XCR1-Positive Cells

To establish scFv binding activity, we evaluated the recognition of the XCR1 receptor and the dependence on protein translocation. These experiments used two CHO cell lines: XCR1^+^ and XCR1^–^. We performed an initial recognition study by incubating the AF647-labeled scFv with CHO cells, followed by flow cytometry analysis. The plots show preferential recognition of the XCR1^+^ but not XCR1^–^ cells (Figure S7).

Protein translocation studies were enabled by the fusion of LF_N_ to the A-chain of diphtheria toxin (LF_N_-DTA), which provided a reporter protein to measure the translocation efficiency based on cell viability. DTA internalization into the cytosol inhibits the elongation factor 2 protein and, in turn, mRNA translation, which leads to cell death. However, DTA cannot internalize in the absence of the B-subunit and, therefore, is not toxic when administered alone. For establishing mechanisms of protein translocation, CHO cells are conventionally treated with 20 nM PA and 10-fold serial dilutions of LF_N_-DTA.^40^

**Figure 4:**
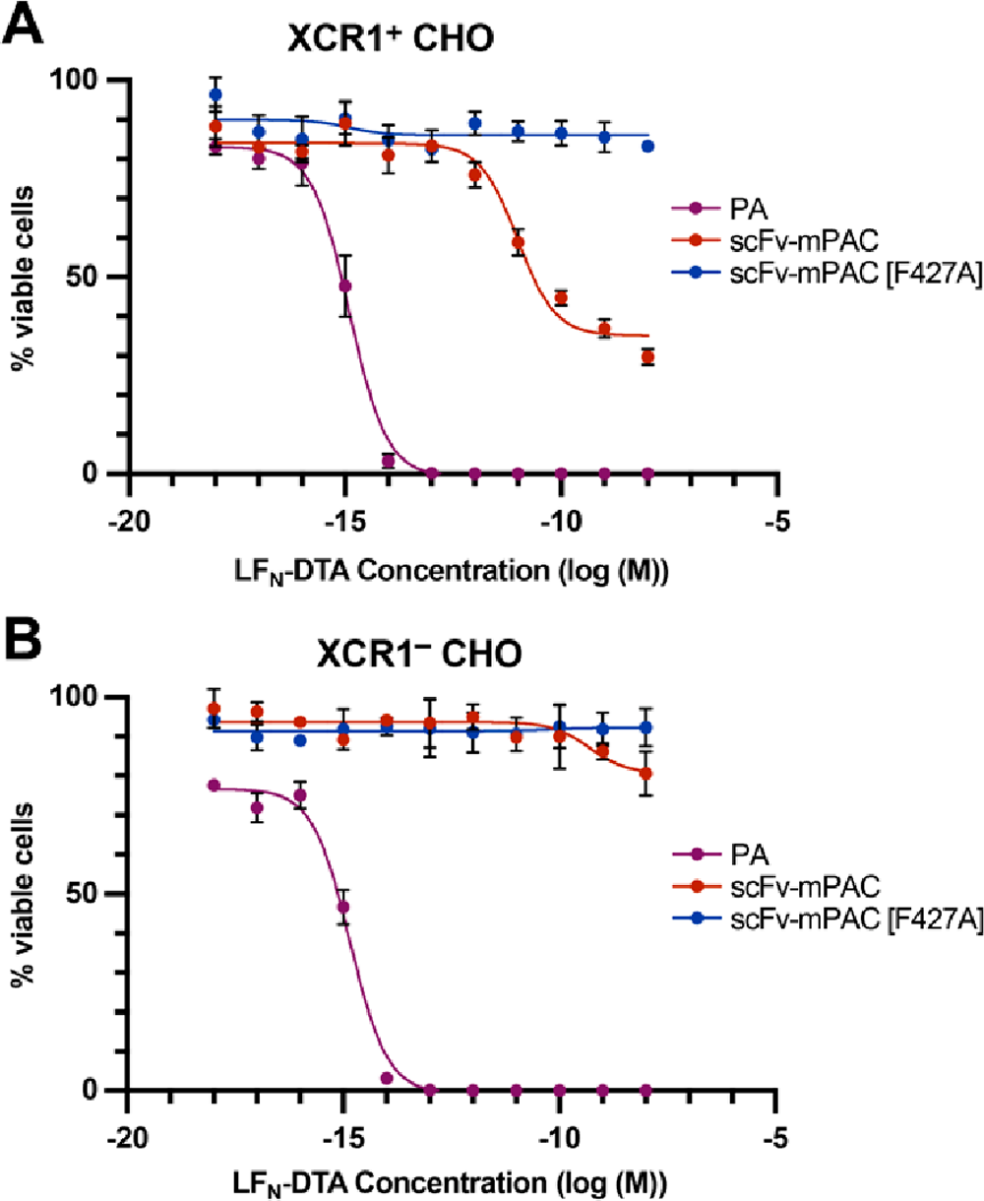
ScFv-directed PA mediates selective protein translocation into XCR1-positive cells. Relative cell viability from the translocated A-chain of diphtheria toxin (DTA) into (A) XCR1^+^ and (B) XCR1^–^ CHO cells. Cells were incubated (72 h) with 10-fold serial dilutions of LF_N_-DTA in the presence of 20 nM PA, scFv-mPAC, or scFv-mPAC[F427A]. Relative viability (% viable cells) was determined by measuring luminescence from a Cell Titer-Glo assay; the viability was normalized to untreated cells. Data represent the mean of three replicate wells ± the standard deviation (s.d.). Data are representative of two independent experiments.

Here, we established that scFv-mPAC exerts translocation by a mechanism that is dependent on the scFv recognition of XCR1 receptors. These experiments also used XCR1^+^ and XCR1^–^ CHO cells (Figure 4). The treatment conditions were based on conventional concentrations, in which cells were treated with 20 nM PA, scFv-mPAC, or scFv-mPAC[F427A] and with 10-fold serial dilutions of LF_N_-DTA. After 72 h, native PA decreased viability for both XCR1^–^ (EC_50_ = 1.4 fM) and XCR1^+^ (EC_50_ = 1.3 fM) cell lines; scFv-mPAC decreased viability only for the XCR1^+^ (EC_50_ = 9.9 pM) but not the XCR1^–^ cells; and scFv-mPAC[F427A] did not decrease viability. Furthermore, treatment with LF_N_-DTA alone, without translocation, did not decrease viability (Figure S8). The absence of toxicity from LF_N_-DTA administered alone and when coadministered with scFv-mPAC[F427A] demonstrates that only translocated LF_N_-DTA decreases viability, rather than the individual PA components.

The viability data from scFv-mPAC are consistent with an XCR1-dependent, PA-mediated translocation mechanism (Figure 4A). This mechanism is dependent on CHO expression of XCR1 receptors, in which the potency is influenced by receptor expression and binding affinity. Although the potency for the XCR1-targeting PA is lower than that of native PA, this decrease in potency may reflect differences in receptor expression or binding affinity. We postulate that the plateau in viability is due to the presence of CHO cell populations that do not contain XCR1 and are therefore not susceptible to scFv binding. Moreover, the scFv-mPAC does not decrease the viability of the XCR1^–^ cells, which further infers that the absence of XCR1 precludes protein translocation (Figure 4B). These observations corroborate the scFv-mPAC proposed mechanisms of XCR1-targeting and receptor-mediated cytosolic delivery into XCR1-positive cells.

### Biodistribution of XCR1-Targeting Constructs in Whole Mice

**Figure 5:**
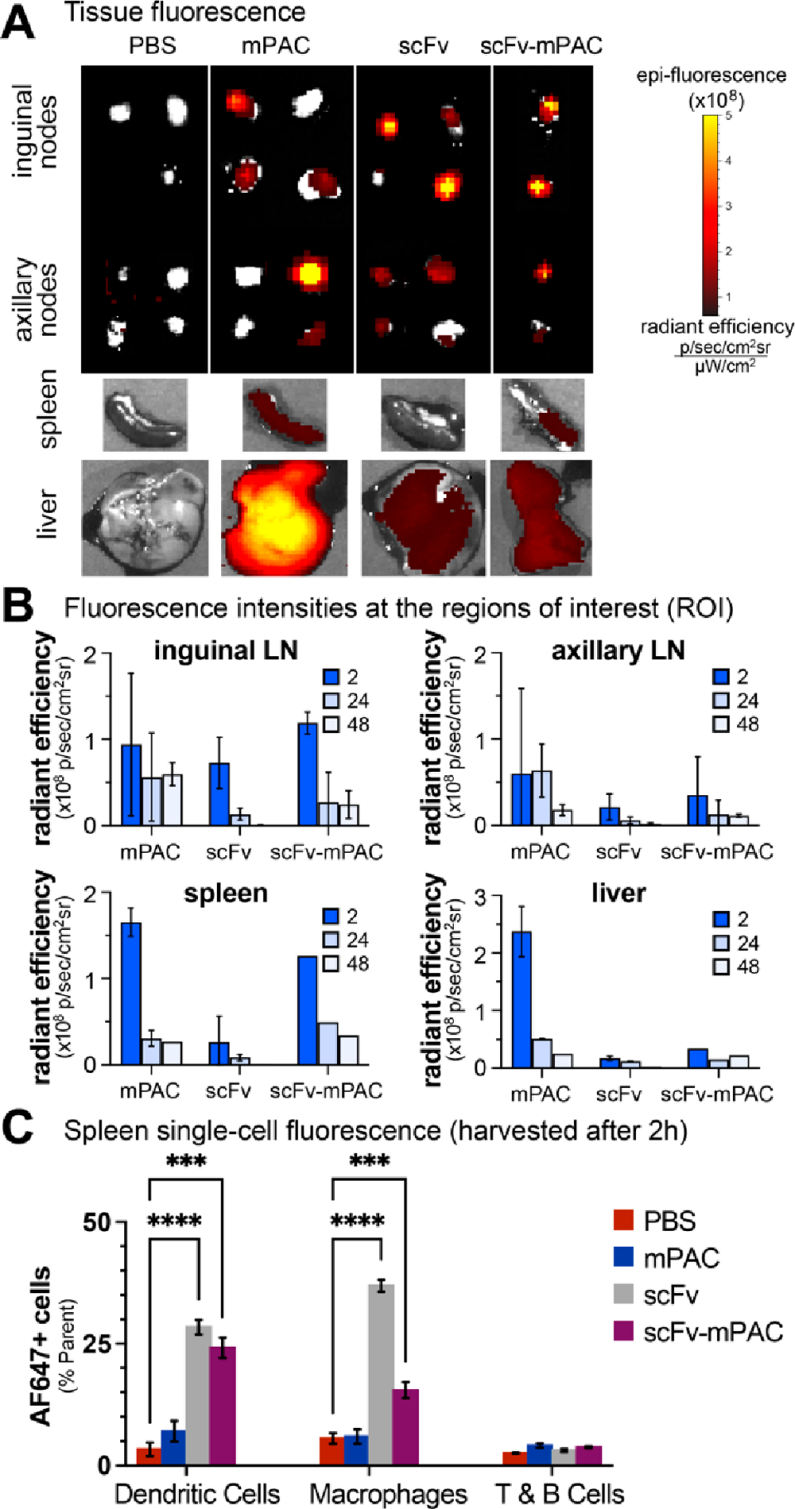
Targeting XCR1 facilitates trafficking to lymph nodes and antigen-presenting cells. (A,C) Time-course analysis of AF647 signal in mouse organs: two mice per time point for mPAC and scFv; one mouse per time point for scFv-mPAC. Mice were treated with 1 nmol of an AF647-labeled construct: mPAC, anti-XCR1 scFv, or anti-XCR1 scFv-mPAC. The constructs were subcutaneously (sc) administered over two equal volume injections, one on each side of the tail base (*n* = 5 mice per group for mPAC and scFv; *n* = 3 mice for scFv-mPAC). (A) Representative images obtained after 2 h using an in vivo imaging system (IVIS), showing AF647 signal from resected lymph nodes (inguinal and axillary), spleen, and liver. Data represent the mean of whole-organ radiant efficiency ± s.d. (B) Quantification of the AF647 signal after 2, 24, and 48 h. (C) Flow cytometry analysis of the AF647 signal after 2 h in single-cell splenocyte populations, including CD8^+^ dendritic cells (CD11c^+^CD8^+^), medullary macrophages (CD11b^+^F4/80^+^), and T and B cells (CD3^+^B220^+^). Data represent the mean of AF647+ cells ± s.d. All data are representative of two independent experiments.

Anti-XCR1 scFv-mPAC trafficking and biodistribution were evaluated in healthy mice using whole-organ ex vivo near-infrared fluorescence (NIRF) imaging. The mPAC, scFv, and scFv-mPAC constructs were conjugated to commercial Alexa-Fluor 647 (AF647) dyes to give mPAC-AF647, scFv-AF647, and scFv-mPAC-AF647, respectively. AF647 (absorption: 650 nm, emission: 665 nm) is a well-characterized fluorochrome used for preclinical NIRF imaging in mice.^41−43^ NIRF imaging was performed at 2, 24, and 48 h post subcutaneous (s.c.) injection with 1 nmol of scFv-AF647, mPAC-AF647, scFv-mPAC-AF647 in C57Bl/6J mice (Figure 5A, B). ScFv-AF647 distributed in a few hours with residual signal remaining after 24 h, accumulating mainly in the inguinal and axillary lymph nodes and spleen. mPAC-AF647 cleared mainly through the liver and was detected for up to 48 h in the inguinal and axillary lymph nodes. The scFv-mPAC-AF647 showed lower uptake in the liver, indicating reduced off-target uptake compared to mPAC-AF647 administered alone and significantly higher trafficking to the inguinal lymph nodes at 2 h postinjection. Furthermore, the uptake of scFv-mPAC-AF647 in the spleen was higher at 24 and 48 h compared to mPAC-AF647 and scFv-AF647 administered alone. Toxicity is a major concern for drug development and these findings suggest that scFv-mPAC has a more favorable pharmacokinetic profile than mPAC alone, with a lower potential for adverse toxicity in the liver.

We also evaluated the single-cell fluorescence of AF647-positive splenocytes at 2 h post s.c. injection using flow cytometry (Figure 5C). These studies showed that a significant fraction of AF647-positive cDC1s were detected after the s.c. injection of scFv (28 ± 1.5%) and scFv-mPAC (24 ± 2.1%) compared to the control vehicles (3 ± 1.4%). In addition, the AF647-positive macrophages were also significantly higher after scFv (37 ± 1.2%) and scFv-mPAC (16 ± 1.7%) injections compared to the controls (6 ± 1.1%). Nonetheless, the AF647-positive T and B cells remained similar in all groups. These findings confirm that anti-XCR1 scFv distributes through mouse lymph nodes and spleen and promotes uptake by CD8^+^ DC.

The specificity of anti-XCR1 scFv was further evaluated with murine splenocytes ex vivo. Single-cell suspensions of murine splenocytes (C57BL/6) were incubated with the scFv, followed by antibody staining and flow cytometry analysis (Figures S9–S11). The data show that the scFv preferentially recognizes CD8^+^ DCs; the data also show nonspecific uptake by medullary macrophages, which are known for phagocytosis of protein antigens.^44^

### Cytosolic Delivery of Antigens into Dendritic Cells Enhances Antigen Immunogenicity

**Figure 6:**
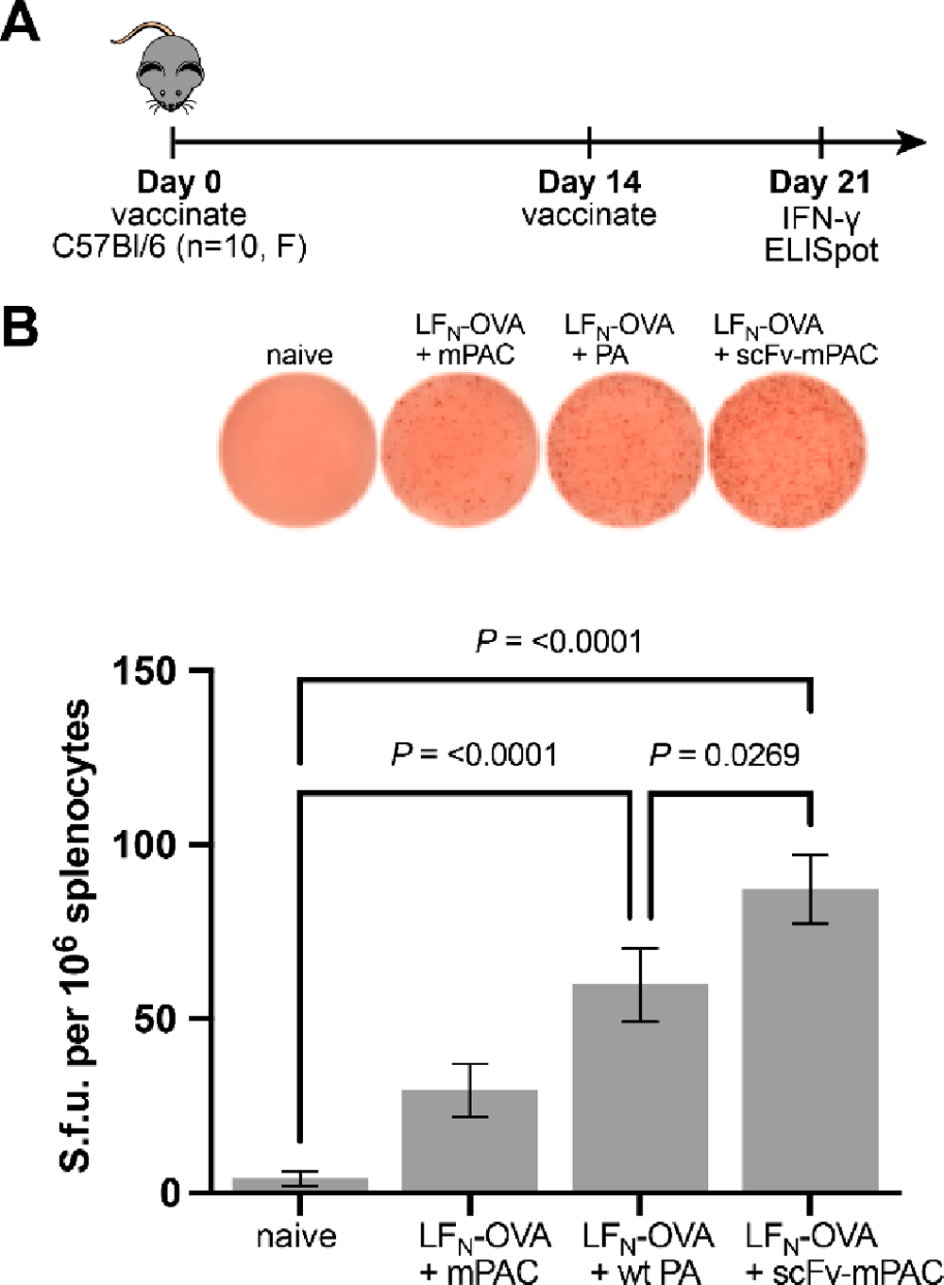
XCR1-targeted intracellular delivery outperforms nontargeted constructs. (A) Mice were s.c. vaccinated with LF_N_-OVA (30 pmol, OVA_252–270_) and c-di-GMP (25 μg), which were coadministered with mPAC, PA, or scFv-mPAC (6 pmol). (B) IFN-γ enzyme-linked immunosorbent spot (ELISpot) data are shown from 7 days after the boost (mean ± SEM; *n* = 10 mice per group). Statistical significance was calculated using a one-way ANOVA with the Fisher’s Least Significant Difference test. Data are compiled from two independent experiments.

PA/LF_N_-mediated antigen delivery confers antigen-specific immunity by a mechanism consistent with MHC class I presentation and the priming of CD8^+^ T cells. We evaluated this immune response in mice using the established epitope of ovalbumin: SIINFEKL (OVA_257–264_).^45,46^ The vaccine comprised a synthetic long epitope peptide: OVA_257–270_. This peptide was incorporated into LF_N_ to give LF_N_-OVA_257–270_ (Table S2; Figure S12) and was s.c. administered to mice with mPAC, PA, or scFv-mPAC (Figure 6A). The c-di-GMP adjuvant, an activator of the STING signaling pathway, was coadministered with the vaccine to enhance the immune response.^47−49^ Mice were sacrificed 7 days after receiving the boost to evaluate IFN-γ release from splenocyte cells, enabling quantitation of antigen-specific CTL priming (Figure 6B).^50,51^ Enzyme-linked immunosorbent spot analysis (ELISpot), after stimulation with the SIINFEKL epitope (Table S3; Figure S13), showed that LF_N_-OVA_252–270_ induced a release of proinflammatory IFN-γ cytokines in all groups. When the LF_N_-OVA_252–270_ vaccine was injected with the anti-XCR1 scFv-mPAC, the immunogenicity increased by ∼32% (436 ± 49 colonies) compared to PA (299 ± 53 colonies) and ∼67% to mPAC (147 ± 38 colonies). This study highlights that targeting XCR1 significantly enhances CTL response in immunocompetent mice.

We also evaluated the mechanism of immunity with additional experiments. MHC class I presentation was evaluated by the treatment of murine DC2.4 cells with PA/LF_N_-OVA_257–264_ (Table S2), followed by flow cytometry analysis of the presented epitope.^52^ The data show prominent MHC class I presentation of the SIINFEKL peptide after 6 h, which decreases after 24 h (Figure S14). Antigen-specific CTL responses were evaluated by MHC tetramer and intracellular cytokine staining (ICS). The data show priming of OVA-specific T cells from the combined treatment with c-di-GMP and PA/LF_N_-OVA_257–264_ but not from the monotherapies with PA or LF_N_-OVA_257–264_ alone (Figure S15). The data show a measurable persistence of this CTL response after four vaccinations (Figure S16), which steadily decreases after each dose due to potential neutralizing antibodies responses that disable the carrier proteins (i.e., PA and LF_N_). These studies further establish that the CTL response is antigen-specific and persists after repeat injections.

### LF_N_-Trp1-gp100/scFv-mPAC Vaccine Is Effective in Mice Bearing B16–F10 Tumors

B16–F10 is a highly aggressive murine cancer model and is widely used for either subcutaneous or metastatic models of melanoma.^53−55^ B16 tumors are known to downregulate class I MHC to limit recognition by cytotoxic T cells.^56,57^ We chose to target two cell-studied tumor associated antigens expressed by B16 tumors, Trp1 and gp100.^58−60^

**Figure 7:**
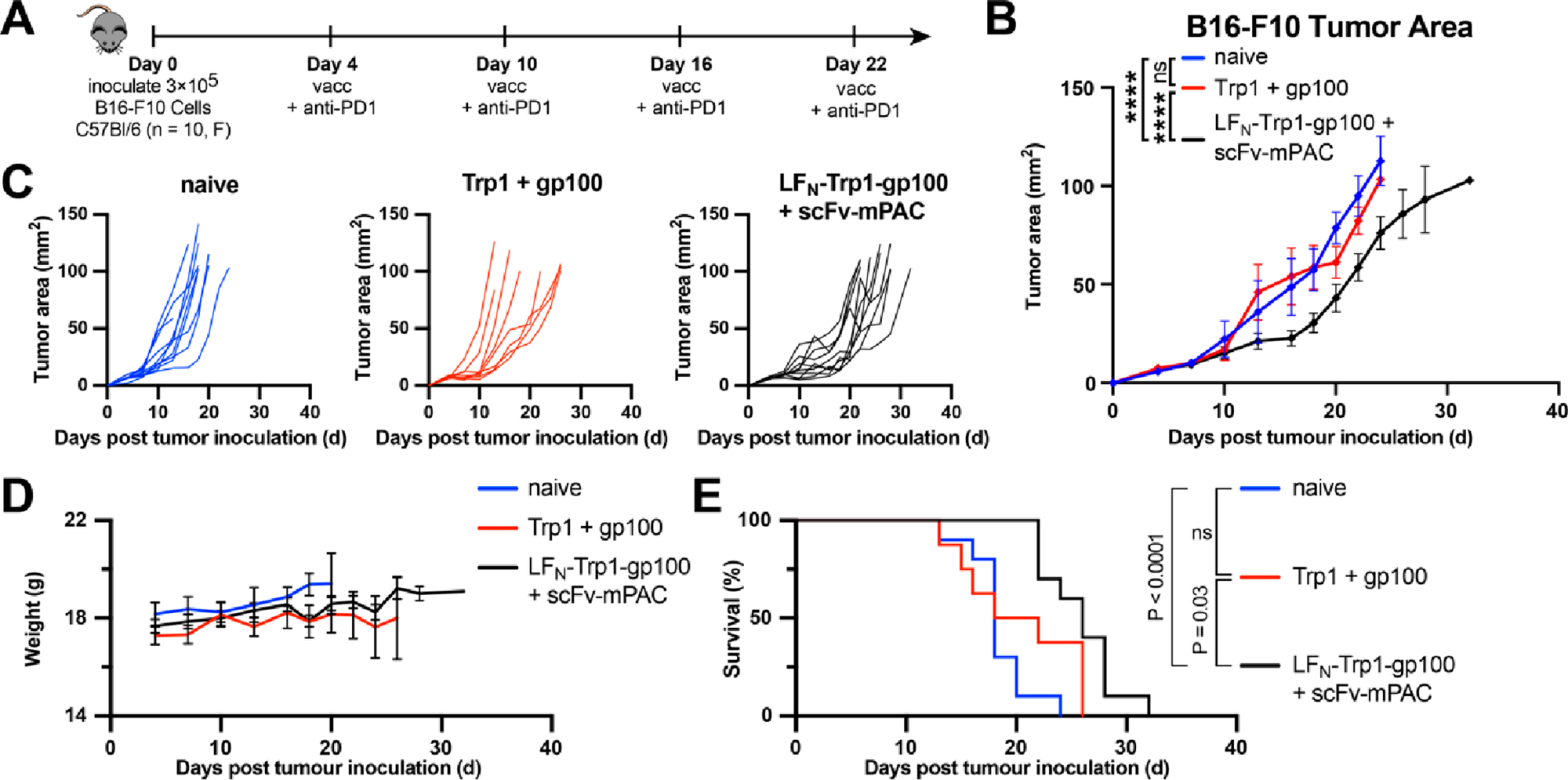
Vaccine efficacy for cancer immunotherapy. (A) Immunization timeline. Mice (F, C57Bl/6, *n* = 10 per group) were s.c. inoculated with 3 × 10^5^ B16–F10 cells (day 0). On days 4, 10, 16, and 22, all mice were intraperitoneally (i.p.) injected with anti-PD1 antibody (200 μg) and were s.c. vaccinated (vacc) with c-di-GMP (25 μg) combined with either Trp1 + gp100 peptides (50 pmol each) or LF_N_-Trp1-gp100 (50 pmol) + scFv-mPAC (10 pmol). (B) Mean tumor growth (mm^2^) per group. Data represent the mean tumor growth ± SEM. Statistical significance was calculated using two-way ANOVA with Tukey test (main effect only model) with multiple mean comparisons on the entire curves, * *P* < 0.05; ** *P* < 0.005; *** *P* < 0.001; and **** *P* < 0.0001. (C) Tumor growth plots for the individual mice over the entire experiment. (D) Body weight. Data represent the mean + SEM (E) Kaplan–Meier percent of survival curves. Statistical significance was calculated using the log-rank Mantel–Cox test. Median survival: 18 days in the naïve group, 20 days in the Trp1 + gp100 group, and 26 days in the LF_N_-Trp1-gp100 + scFv-mPAC group.

In the present study, we aimed to evaluate the peptide antigen Trp1-gp100 (Table S2; Figure S17) when conjugated to LF_N_ (Figure S18), followed by translocation into DCs. We envisioned that the anti-XCR1 scFv-mPAC would facilitate DC maturation and recruit CD8^+^ T cells when compared with the unconjugated antigens (Table S3; Figures S19, S20). We hypothesized the dual activity of the melanoma antigens would increase the immunogenic response against B16–F10 tumors. Given the aggressive nature of the B16 model, we combined vaccination with anti-PD-1 checkpoint blockade to further amplify antitumor activity. The efficacy of the LF_N_-Trp1-gp100/scFv-mPAC vaccine was assessed in C57Bl/6J immunocompetent mice bearing subcutaneous B16–F10 tumors (Figure 7A). Tumor growth was monitored over the entire experiment (Figure 7B). On days 13 and 16 post-tumor induction, significant inhibition of tumor growth was observed in the LF_N_-Trp1-gp100/scFv-mPAC group but not in the naïve group or the Trp1 + gp100 group. After day 16, the mice vaccinated with LF_N_-Trp1-gp100/scFv-mPAC showed inhibited tumor growth, compared to the controls, until the end of the experiment (*p* < 0.0001).

Tumor growth curves upon treatment with LF_N_-Trp1-gp100/scFv-mPAC show a plateau from day 10 to 20, with tumor growth returning to a similar rate as the control groups approximately after day 20 (Figure 7C). No significant vaccine-related toxicity was noticed after the four rounds of vaccination, as neither loss of appetite, loss of weight, reduction of socialization, nor change in behavior was observed (Figure 7D). Kaplan–Meier analysis indicates that the LF_N_-Trp1-gp100/scFv-mPAC vaccine significantly prolongs the survival of mice, with a median survival of 26 days, versus 18 days for the naive (*P* < 0.0001) and 20 days for the Trp1 + gp100 (*P* = 0.03) groups (Figure 7E). Both tumor growth and survival data highlight that B16–F10 cancer progression was significantly delayed from about a week after vaccination with LF_N_-Trp1-gp100/scFv-mPAC.

Nonetheless, treatment with LF_N_-Trp1-gp100/scFv-mPAC showed wide variation in tumor growth and relapse after day 20, indicating an incomplete response. Potential resistance to the PA/LF_N_ carrier proteins may explain the tumor relapse after the third vaccine injection. Similar observations have been made in a recent study, following vaccination of mice bearing B16–F10 tumors with TTR-Trp1-gp100 peptides.^61^ Although anti-PD1 antibodies were administered concomitantly with the vaccine, these treatments may have been insufficient to obtain complete inhibition of ICB as some animals may have become unresponsive due to potential mutations occurring in the IFN-γ-JAK-STAT signaling pathway.^62−64^ While the coadministration of the STING analog adjuvant c-di-GMP should increase the sensitivity for anti-PD-1 antibodies,^47−49^ melanoma-bearing mice can undertake adaptive ICB resistance. Indeed, B16–F10 tumors are described to have genetic or acquired resistance mechanisms to vaccines and drugs, and are therefore challenging to treat, thereby explaining that no animal was cured during the therapy study.^65^

After day 10, tumors in the control groups showed hyperpigmentation, followed by necrosis, due to postinflammatory overaccumulation of eumelanin. However, in the LF_N_-Trp1-gp100/scFv-mPAC group, we observed that, even after day 20, the tumors were non- or little-pigmented, and, after the fourth vaccine injection, there was a loss of pigmentation and change in tumor phenotype (Figure S21). Melanogenesis is regulated by an array of inflammation signals, including cytokines, interleukins, tumor necrosis factor (TNF), or prostaglandin E2, among others.^66^ In B16–F10 tumors, the production of melanin can be inhibited by IFN-γ which blocks the maturation of the melanosome and upregulates the phosphorylation of the STAT1 signaling pathway, both resulting in cell apoptosis.^67,68^ The hypopigmentation of tumors in the vaccine group is thereby consistent with the abrogation of tumor growth and improvement of survival. These outcomes suggest that the dual translocation of Trp1/gp100 into DCs using the LF_N_/scFv-mPAC vaccine induced overexpression of IFN-γ and therefore induced a strong reduction in tumor growth.

## Discussion

Therapeutic cancer vaccines rely on administering large amounts of specific tumor antigens to activate DCs and induce a durable CTL response.^2^ Despite considerable efforts, the first generations of therapeutic cancer vaccines failed to elicit sufficient T cell activation and long-lasting immunity, mainly due to insufficient delivery and/or the choice of tumor-specific antigens.^69−71^ Subsequent cancer vaccine research has focused on improving the nature and selection of target antigens, including through the identification of immunogenic tumor mutations and the assessment of epitope binding affinity to HLA alleles.^72−74^ Tumor antigens that are unique to each patient, called neoantigens, have also motivated the development of personalized cancer vaccines.^75^ A major challenge for developing next-generation therapeutic vaccines is engineering more specific and effective delivery platforms of these tumor antigens.

In previous studies, we engineered cancer-targeting mPAC constructs to translocate cytotoxic payloads.^34,76^ Here, we developed a DC-targeting delivery system for immunogenic epitopes. The delivery system comprises an anti-XCR1 scFv conjugated to mPAC, which is a nontoxic triple mutant anthrax protective antigen protein. Administering the scFv-mPAC with an antigen-conjugated LF_N_ activated in vivo immune responses through the recruitment of CD8^+^ T cells and demonstrates that the scFv-mPAC/LF_N_ system targets DCs with tumor-specific antigens. In mice, s.c. administration of the DC-targeting platform showed trafficking through the lymphatic system and a significant activation of CD8^+^ T cells in the spleen. Moreover, the administration of scFv-mPAC with an LF_N_-conjugated antigen OVA_252–270_ increased the immunogenicity in splenocytes by 32% compared to the nontargeting of XCR1 antigen.

Recently, a T cell engager medication comprising a bispecific fusion anti-CD3 protein targeting gp100 membrane antigen (Tebentafusp) has shown potent antitumor response with significant improvement of overall survival at 1 year in patients with metastatic refractory melanoma^60,77^ and HLA-A*02:01-positive uveal melanoma patients^78^ for which it gained FDA approval in 2022. We have therefore selected gp100, but also Trp1 tumor-specific antigens, for the construction of a dual LF_N_ conjugate and assessed DCs targeting efficacy in an aggressive melanoma model. Administering scFv-mPAC in combination with LF_N_-Trp1-gp100 in B16–F10 murine melanoma demonstrated a significant abrogation of tumor growth and tumor depigmentation, which was accompanied by a significant extension of survival (26 days compared to 18 and 20 days for the controls).

## Conclusion

The studies described in this paper embody a proof-of-concept cancer vaccine that utilizes the anthrax PA/LF_N_ translocation mechanism for targeting and loading cDC1s with tumor antigens to prime CD8^+^ T cells. Our studies show that PA/LF_N_ can be engineered to target XCR1^+^ DCs, translocate antigenic cargo, and enhance priming of antigen-specific CTL responses. The scFv-mPAC/LF_N_-Trp1-gp100 vaccination against B16–F10 melanoma demonstrates that this platform inhibits tumor cell growth, even of aggressive cancers. Our results validate the importance of developing cancer immunotherapies that target DCs. We are currently investigating additional immunotherapy platforms that target DCs and will report our findings in due course.

## Supporting Information Available

Materials, methods, analytical, biological, and animal data (PDF)

## References

[ref1] Milling L.; Zhang Y.; Irvine D. J. Delivering safer immunotherapies for cancer. Adv. Drug Deliv Rev. 2017, 114, 79–101. 10.1016/j.addr.2017.05.011. 28545888 PMC5647831

[ref2] Saxena M.; van der Burg S. H.; Melief C. J. M.; Bhardwaj N. Therapeutic cancer vaccines. Nat. Rev. Cancer. 2021, 21 (6), 360–78. 10.1038/s41568-021-00346-0. 33907315

[ref3] Waldman A. D.; Fritz J. M.; Lenardo M. J. A guide to cancer immunotherapy: from T cell basic science to clinical practice. Nat. Rev. Immunol. 2020, 20 (11), 651–68. 10.1038/s41577-020-0306-5. 32433532 PMC7238960

[ref4] Sellars M. C.; Wu C. J.; Fritsch E. F. Cancer vaccines: Building a bridge over troubled waters. Cell. 2022, 185 (15), 2770–88. 10.1016/j.cell.2022.06.035. 35835100 PMC9555301

[ref5] Miao L.; Zhang Y.; Huang L. mRNA vaccine for cancer immunotherapy. Mol. Cancer. 2021, 20 (1), 41 10.1186/s12943-021-01335-5. 33632261 PMC7905014

[ref6] Liu W.; Tang H.; Li L.; Wang X.; Yu Z.; Li J. Peptide-based therapeutic cancer vaccine: Current trends in clinical application. Cell Prolif. 2021, 54 (5), e13025 10.1111/cpr.13025. 33754407 PMC8088465

[ref7] Satpathy A. T.; Wu X.; Albring J. C.; Murphy K. M. Re(de)fining the dendritic cell lineage. Nature Immunology. 2012, 13 (12), 1145–54. 10.1038/ni.2467. 23160217 PMC3644874

[ref8] Macri C.; Dumont C.; Johnston A. P.; Mintern J. D. Targeting Dendritic Cells: a Promising Strategy to Improve Vaccine Effectiveness. Clinical & translational immunology. 2016, 5 (3), e66 10.1038/cti.2016.6. 27217957 PMC4815026

[ref9] Apostolopoulos V.; Thalhammer T.; Tzakos A. G.; Stojanovska L. Targeting antigens to dendritic cell receptors for vaccine development. Journal of drug delivery. 2013, 2013, 1 10.1155/2013/869718. PMC3817681 24228179

[ref10] Joffre O. P.; Segura E.; Savina A.; Amigorena S. Cross-presentation by dendritic cells. Nat. Rev. Immunol. 2012, 12 (8), 557–69. 10.1038/nri3254. 22790179

[ref11] Palucka K.; Banchereau J. Cancer Immunotherapy via Dendritic Cells. Nat. Rev. Cancer. 2012, 12 (4), 265–77. 10.1038/nrc3258. 22437871 PMC3433802

[ref12] Merad M.; Sathe P.; Helft J.; Miller J.; Mortha A. The dendritic cell lineage: ontogeny and function of dendritic cells and their subsets in the steady state and the inflamed setting. Annual review of immunology. 2013, 31, 563–604. 10.1146/annurev-immunol-020711-074950. PMC3853342 23516985

[ref13] Wylie B.; Read J.; Buzzai A. C.; Wagner T.; Troy N.; Syn G.; Stone S. R.; Foley B.; Bosco A.; Cruickshank M. N.; Waithman J. CD8(+)XCR1(neg) Dendritic Cells Express High Levels of Toll-Like Receptor 5 and a Unique Complement of Endocytic Receptors. Frontiers Immunol. 2019, 9, 2990 10.3389/fimmu.2018.02990. PMC6343586 30700986

[ref14] Bachem A.; Hartung E.; Guttler S.; Mora A.; Zhou X.; Hegemann A.; Plantinga M.; Mazzini E.; Stoitzner P.; Gurka S.; Henn V.; Mages H. W.; Kroczek R. A. Expression of XCR1 Characterizes the Batf3-Dependent Lineage of Dendritic Cells Capable of Antigen Cross-Presentation. Frontiers Immunol. 2012, 3, 214 10.3389/fimmu.2012.00214. PMC3399095 22826713

[ref15] Tullett K. M.; Lahoud M. H.; Radford K. J. Harnessing Human Cross-Presenting CLEC9A(+)XCR1(+) Dendritic Cells for Immunotherapy. Frontiers Immunol. 2014, 5, 239 10.3389/fimmu.2014.00239. PMC4033245 24904587

[ref16] Dorner B. G.; Dorner M. B.; Zhou X.; Opitz C.; Mora A.; Guttler S.; Hutloff A.; Mages H. W.; Ranke K.; Schaefer M.; Jack R. S.; Henn V.; Kroczek R. A. Selective expression of the chemokine receptor XCR1 on cross-presenting dendritic cells determines cooperation with CD8+ T cells. Immunity 2009, 31 (5), 823–33. 10.1016/j.immuni.2009.08.027. 19913446

[ref17] Murphy T. L.; Murphy K. M. Dendritic cells in cancer immunology. Cell Mol. Immunol. 2022, 19 (1), 3–13. 10.1038/s41423-021-00741-5. 34480145 PMC8752832

[ref18] Bowman-Kirigin J. A.; Desai R.; Saunders B. T.; Wang A. Z.; Schaettler M. O.; Liu C. J.; Livingstone A. J.; Kobayashi D. K.; Durai V.; Kretzer N. M.; Zipfel G. J.; Leuthardt E. C.; Osbun J. W.; Chicoine M. R.; Kim A. H.; Murphy K. M.; Johanns T. M.; Zinselmeyer B. H.; Dunn G. P. The Conventional Dendritic Cell 1 Subset Primes CD8+ T Cells and Traffics Tumor Antigen to Drive Antitumor Immunity in the Brain. Cancer immunology research. 2023, 11 (1), 20–37. 10.1158/2326-6066.CIR-22-0098. 36409838 PMC10725570

[ref19] Barry K. C.; Hsu J.; Broz M. L.; Cueto F. J.; Binnewies M.; Combes A. J.; Nelson A. E.; Loo K.; Kumar R.; Rosenblum M. D.; Alvarado M. D.; Wolf D. M.; Bogunovic D.; Bhardwaj N.; Daud A. I.; Ha P. K.; Ryan W. R.; Pollack J. L.; Samad B.; Asthana S.; Chan V.; Krummel M. F. A natural killer-dendritic cell axis defines checkpoint therapy-responsive tumor microenvironments. Nat. Med. 2018, 24 (8), 1178–91. 10.1038/s41591-018-0085-8. 29942093 PMC6475503

[ref20] Michea P.; Noel F.; Zakine E.; Czerwinska U.; Sirven P.; Abouzid O.; Goudot C.; Scholer-Dahirel A.; Vincent-Salomon A.; Reyal F.; Amigorena S.; Guillot-Delost M.; Segura E.; Soumelis V. Adjustment of dendritic cells to the breast-cancer microenvironment is subset specific. Nat. Immunol. 2018, 19 (8), 885–97. 10.1038/s41590-018-0145-8. 30013147

[ref21] Fossum E.; Tesfaye D. Y.; Bobic S.; Gudjonsson A.; Braathen R.; Lahoud M. H.; Caminschi I.; Bogen B. Targeting Antigens to Different Receptors on Conventional Type 1 Dendritic Cells Impacts the Immune Response. Journal of immunology 2020, 205 (3), 661–73. 10.4049/jimmunol.1901119. 32591401

[ref22] Hartung E.; Becker M.; Bachem A.; Reeg N.; Jakel A.; Hutloff A.; Weber H.; Weise C.; Giesecke C.; Henn V.; Gurka S.; Anastassiadis K.; Mages H. W.; Kroczek R. A. Induction of potent CD8 T cell cytotoxicity by specific targeting of antigen to cross-presenting dendritic cells in vivo via murine or human XCR1. Journal Immunol. 2015, 194 (3), 1069–79. 10.4049/jimmunol.1401903. 25520399

[ref23] Yan Z.; Wu Y.; Du J.; Li G.; Wang S.; Cao W.; Zhou X.; Wu C.; Zhang D.; Jing X.; Li Y.; Wang H.; Gao Y.; Qi Y. A Novel Peptide Targeting Clec9a on Dendritic Cell for Cancer Immunotherapy. Oncotarget 2016, 7 (26), 40437–50. 10.18632/oncotarget.9624. 27250027 PMC5130018

[ref24] Gros M.; Amigorena S. Regulation of Antigen Export to the Cytosol During Cross-Presentation. Frontiers Immunol. 2019, 10 (41), 1 10.3389/fimmu.2019.00041. PMC6360170 30745902

[ref25] Blum J. S.; Wearsch P. A.; Cresswell P. Pathways of antigen processing. Annual review of immunology. 2013, 31, 443–73. 10.1146/annurev-immunol-032712-095910. PMC4026165 23298205

[ref26] Allahyari H.; Heidari S.; Ghamgosha M.; Saffarian P.; Amani J. Immunotoxin: A new tool for cancer therapy. Tumor Biology. 2017, 39 (2), 101042831769222 10.1177/1010428317692226. 28218037

[ref27] Ballard J. D.; Collier R. J.; Starnbach M. N. Anthrax Toxin-Mediated Delivery of a Cytotoxic T-Cell Epitope in vivo. Proc. Natl. Acad. Sci. U. S. A. 1996, 93 (22), 12531–4. 10.1073/pnas.93.22.12531. 8901616 PMC38026

[ref28] Ballard J. D.; Doling A. M.; Beauregard K.; Collier R. J.; Starnbach M. N. Anthrax Toxin-Mediated Delivery In Vivo and In Vitro of a Cytotoxic T-Lymphocyte Epitope from Ovalbumin. Infect. Immun. 1998, 66 (2), 615–9. 10.1128/IAI.66.2.615-619.1998. 9453617 PMC107948

[ref29] Rabideau A. E.; Pentelute B. L. Delivery of Non-Native Cargo into Mammalian Cells Using Anthrax Lethal Toxin. ACS Chem. Biol. 2016, 11 (6), 1490–501. 10.1021/acschembio.6b00169. 27055654

[ref30] Mechaly A.; McCluskey A. J.; Collier R. J. Changing the receptor specificity of anthrax toxin. mBio 2012, 3 (3), 1 10.1128/mBio.00088-12. PMC3569862 22550037

[ref31] McCluskey A. J.; Collier R. J. Receptor-directed chimeric toxins created by sortase-mediated protein fusion. Molecular cancer therapeutics. 2013, 12 (10), 2273–81. 10.1158/1535-7163.MCT-13-0358. 23945077 PMC3795991

[ref32] McCluskey A. J.; Olive A. J.; Starnbach M. N.; Collier R. J. Targeting HER2-Positive Cancer Cells with Receptor-Redirected Anthrax Protective Antigen. Mol. Oncol. 2013, 7, 440–51. 10.1016/j.molonc.2012.12.003. 23290417 PMC3621010

[ref33] Jack S.; Madhivanan K.; Ramadesikan S.; Subramanian S.; Edwards D. F.; Elzey B. D.; Dhawan D.; McCluskey A.; Kischuk E. M.; Loftis A. R.; Truex N.; Santos M.; Lu M.; Rabideau A.; Pentelute B.; Collier J.; Kaimakliotis H.; Koch M.; Ratliff T. L.; Knapp D. W.; Aguilar R. C. A novel, safe, fast and efficient treatment for Her2-positive and negative bladder cancer utilizing an EGF-anthrax toxin chimera. Int. J. Cancer. 2020, 146 (2), 449–60. 10.1002/ijc.32719. 31584195 PMC10303116

[ref34] Loftis A. R.; Santos M. S.; Truex N. L.; Biancucci M.; Satchell K. J. F.; Pentelute B. L. Anthrax protective antigen retargeted with single-chain variable fragments delivers enzymes to pancreatic cancer cells. ChemBioChem 2020, 21 (19), 2772–6. 10.1002/cbic.202000201. 32369652 PMC7541672

[ref35] Lu Z.; Truex N. L.; Melo M. B.; Cheng Y.; Li N.; Irvine D. J.; Pentelute B. L. IgG-Engineered protective antigen for cytosolic delivery of proteins into cancer cells. ACS Cent. Sci. 2021, 7 (2), 365–78. 10.1021/acscentsci.0c01670. 33655074 PMC7908032

[ref36] Kroczek R. Antibodies to the chemokine receptor xcr1. European Patent EP 2 641 915 A1, 2013.

[ref37] Rosovitz M. J.; Schuck P.; Varughese M.; Chopra A. P.; Mehra V.; Singh Y.; McGinnis L. M.; Leppla S. H. Alanine-scanning mutations in domain 4 of anthrax toxin protective antigen reveal residues important for binding to the cellular receptor and to a neutralizing monoclonal antibody. J. Biol. Chem. 2003, 278 (33), 30936–44. 10.1074/jbc.M301154200. 12771151

[ref38] Mourez M.; Yan M.; Lacy D. B.; Dillon L.; Bentsen L.; Marpoe A.; Maurin C.; Hotze E.; Wigelsworth D.; Pimental R. A.; Ballard J. D.; Collier R. J.; Tweten R. K. Mapping Dominant-Negative Mutations of Anthrax Protective Antigen by Scanning Mutagenesis. Proc. Natl. Acad. Sci. U. S. A. 2003, 100 (24), 13803–8. 10.1073/pnas.2436299100. 14623961 PMC283502

[ref39] Miller C. J.; Elliott J. L.; Collier R. J. Anthrax protective antigen: prepore-to-pore conversion. Biochemistry. 1999, 38 (32), 10432–41. 10.1021/bi990792d. 10441138

[ref40] Milne J. C.; Blanket S. R.; Hanna P. C.; Collier R. J. Protective antigen-binding domain of anthrax lethal factor mediates translocation of a heterologous protein fused to its amino- or carboxy-terminus. Mol. Microbiol. 1995, 15 (4), 661–6. 10.1111/j.1365-2958.1995.tb02375.x. 7783638

[ref41] Arlauckas S. P.; Garris C. S.; Kohler R. H.; Kitaoka M.; Cuccarese M. F.; Yang K. S.; Miller M. A.; Carlson J. C.; Freeman G. J.; Anthony R. M.; Weissleder R.; Pittet M. J. In vivo imaging reveals a tumor-associated macrophage-mediated resistance pathway in anti-PD-1 therapy. Sci. Transl. Med. 2017, 9 (389), 1 10.1126/scitranslmed.aal3604. PMC5734617 28490665

[ref42] Gupta P.; Wentland J. A.; Leal M.; Ma D.; Roach R.; Esparza A.; King L.; Spilker M. E.; Bagi C.; Winkelmann C. T.; Giddabasappa A. Assessment of near-infrared fluorophores to study the biodistribution and tumor targeting of an IL13 receptor alpha2 antibody by fluorescence molecular tomography. Oncotarget 2017, 8 (34), 57231–45. 10.18632/oncotarget.19569. 28915667 PMC5593638

[ref43] Refaat A.; Yap M. L.; Pietersz G.; Walsh A. P. G.; Zeller J.; Del Rosal B.; Wang X.; Peter K. In vivo fluorescence imaging: success in preclinical imaging paves the way for clinical applications. J. Nanobiotechnol. 2022, 20 (1), 450 10.1186/s12951-022-01648-7. PMC9571426 36243718

[ref44] Gray E. E.; Cyster J. G. Lymph node macrophages. J. Innate Immun. 2012, 4 (5–6), 424–36. 10.1159/000337007. 22488251 PMC3574571

[ref45] Beck L.; Spiegelberg H. L. The polyclonal and antigen-specific IgE and IgG subclass response of mice injected with ovalbumin in alum or complete Freund’s adjuvant. Cell Immunol. 1989, 123 (1), 1–8. 10.1016/0008-8749(89)90263-3. 2776217

[ref46] Ke Y.; Li Y.; Kapp J. A. Ovalbumin injected with complete Freund’s adjuvant stimulates cytolytic responses. Eur. J. Immunol. 1995, 25 (2), 549–53. 10.1002/eji.1830250237. 7875219

[ref47] Burdette D. L.; Monroe K. M.; Sotelo-Troha K.; Iwig J. S.; Eckert B.; Hyodo M.; Hayakawa Y.; Vance R. E. STING is a direct innate immune sensor of cyclic di-GMP. Nature. 2011, 478 (7370), 515–8. 10.1038/nature10429. 21947006 PMC3203314

[ref48] Wang Z.; Celis E. STING activator c-di-GMP enhances the anti-tumor effects of peptide vaccines in melanoma-bearing mice. Cancer immunology, immunotherapy: CII. 2015, 64 (8), 1057–66. 10.1007/s00262-015-1713-5. 25986168 PMC4648249

[ref49] Chelvanambi M.; Fecek R. J.; Taylor J. L.; Storkus W. J. STING agonist-based treatment promotes vascular normalization and tertiary lymphoid structure formation in the therapeutic melanoma microenvironment. J. Immunother Cancer 2021, 9 (2), e001906 10.1136/jitc-2020-001906. 33526609 PMC7852948

[ref50] Power C. A.; Grand C. L.; Ismail N.; Peters N. C.; Yurkowski D. P.; Bretscher P. A. A valid ELISPOT assay for enumeration of ex vivo, antigen-specific, IFNgamma-producing T cells. J. Immunol Methods. 1999, 227 (1–2), 99–107. 10.1016/S0022-1759(99)00074-5. 10485258

[ref51] Asai T.; Storkus W. J.; Whiteside T. L. Evaluation of the modified ELISPOT assay for gamma interferon production in cancer patients receiving antitumor vaccines. Clin. Diagn. Lab. Immunol. 2000, 7 (2), 145–54. 10.1128/CDLI.7.2.145-154.2000. 10702485 PMC95841

[ref52] Porgador A.; Yewdell J. W.; Deng Y.; Bennink J. R.; Germain R. N. Localization, quantitation, and in situ detection of specific peptide-MHC class I complexes using a monoclonal antibody. Immunity. 1997, 6 (6), 715–26. 10.1016/S1074-7613(00)80447-1. 9208844

[ref53] Gregg R. K. Model Systems for the Study of Malignant Melanoma. Methods Mol. Biol. 2021, 2265, 1–21. 10.1007/978-1-0716-1205-7_1. 33704702

[ref54] Giavazzi R.; Decio A. Syngeneic murine metastasis models: B16 melanoma. Methods Mol. Biol. 2014, 1070, 131–40. 10.1007/978-1-4614-8244-4_10. 24092437

[ref55] Overwijk W. W.; Restifo N. P. B16 as a Mouse Model for Human Melanoma. Curr. Protoc. Immunol. 2000, 1 10.1002/0471142735.im2001s39. PMC2763508 18432774

[ref56] Bradley S. D.; Chen Z.; Melendez B.; Talukder A.; Khalili J. S.; Rodriguez-Cruz T.; Liu S.; Whittington M.; Deng W.; Li F.; Bernatchez C.; Radvanyi L. G.; Davies M. A.; Hwu P.; Lizee G. BRAFV600E Co-opts a Conserved MHC Class I Internalization Pathway to Diminish Antigen Presentation and CD8+ T-cell Recognition of Melanoma. Cancer Immunol. Res. 2015, 3 (6), 602–9. 10.1158/2326-6066.CIR-15-0030. 25795007 PMC4457616

[ref57] Kameyama K.; Vieira W. D.; Tsukamoto K.; Law L. W.; Hearing V. J. Differentiation and the tumorigenic and metastatic phenotype of murine melanoma cells. Int. J. Cancer 1990, 45 (6), 1151–8. 10.1002/ijc.2910450627. 2161802

[ref58] Jimenez-Cervantes C.; Martinez-Esparza M.; Solano F.; Lozano J. A.; Garcia-Borron J. C. Molecular interactions within the melanogenic complex: formation of heterodimers of tyrosinase and TRP1 from B16 mouse melanoma. Biochem. Biophys. Res. Commun. 1998, 253 (3), 761–7. 10.1006/bbrc.1998.9817. 9918801

[ref59] Dougan S. K.; Dougan M.; Kim J.; Turner J. A.; Ogata S.; Cho H. I.; Jaenisch R.; Celis E.; Ploegh H. L. Transnuclear TRP1-specific CD8 T cells with high or low affinity TCRs show equivalent antitumor activity. Cancer Immunol. Res. 2013, 1 (2), 99–111. 10.1158/2326-6066.CIR-13-0047. 24459675 PMC3895912

[ref60] Middleton M. R.; McAlpine C.; Woodcock V. K.; Corrie P.; Infante J. R.; Steven N. M.; Evans T. R. J.; Anthoney A.; Shoushtari A. N.; Hamid O.; Gupta A.; Vardeu A.; Leach E.; Naidoo R.; Stanhope S.; Lewis S.; Hurst J.; O’Kelly I.; Sznol M. Tebentafusp, A TCR/Anti-CD3 Bispecific Fusion Protein Targeting gp100, Potently Activated Antitumor Immune Responses in Patients with Metastatic Melanoma. Clin. Cancer Res. 2020, 26 (22), 5869–78. 10.1158/1078-0432.CCR-20-1247. 32816891 PMC9210997

[ref61] Mehta N. K.; Pradhan R. V.; Soleimany A. P.; Moynihan K. D.; Rothschilds A. M.; Momin N.; Rakhra K.; Mata-Fink J.; Bhatia S. N.; Wittrup K. D.; Irvine D. J. Pharmacokinetic Tuning of Protein-Antigen Fusions Enhances the Immunogenicity of T-Cell Vaccines. Nat. Biomed Eng. 2020, 4 (6), 636–48. 10.1038/s41551-020-0563-4. 32483299 PMC7575059

[ref62] Galvani E.; Mundra P. A.; Valpione S.; Garcia-Martinez P.; Smith M.; Greenall J.; Thakur R.; Helmink B.; Andrews M. C.; Boon L.; Chester C.; Gremel G.; Hogan K.; Mandal A.; Zeng K.; Banyard A.; Ashton G.; Cook M.; Lorigan P.; Wargo J. A.; Dhomen N.; Marais R. Stroma remodeling and reduced cell division define durable response to PD-1 blockade in melanoma. Nat. Commun. 2020, 11 (1), 853 10.1038/s41467-020-14632-2. 32051401 PMC7015935

[ref63] Zaretsky J. M.; Garcia-Diaz A.; Shin D. S.; Escuin-Ordinas H.; Hugo W.; Hu-Lieskovan S.; Torrejon D. Y.; Abril-Rodriguez G.; Sandoval S.; Barthly L.; Saco J.; Homet Moreno B.; Mezzadra R.; Chmielowski B.; Ruchalski K.; Shintaku I. P.; Sanchez P. J.; Puig-Saus C.; Cherry G.; Seja E.; Kong X.; Pang J.; Berent-Maoz B.; Comin-Anduix B.; Graeber T. G.; Tumeh P. C.; Schumacher T. N.; Lo R. S.; Ribas A. Mutations Associated with Acquired Resistance to PD-1 Blockade in Melanoma. New Engl. J. Med. 2016, 375 (9), 819–29. 10.1056/NEJMoa1604958. 27433843 PMC5007206

[ref64] Nguyen T. T.; Ramsay L.; Ahanfeshar-Adams M.; Lajoie M.; Schadendorf D.; Alain T.; Watson I. R. Mutations in the IFNgamma-JAK-STAT Pathway Causing Resistance to Immune Checkpoint Inhibitors in Melanoma Increase Sensitivity to Oncolytic Virus Treatment. Clin. Cancer Res. 2021, 27 (12), 3432–42. 10.1158/1078-0432.CCR-20-3365. 33593882

[ref65] Patton E. E.; Mueller K. L.; Adams D. J.; Anandasabapathy N.; Aplin A. E.; Bertolotto C.; Bosenberg M.; Ceol C. J.; Burd C. E.; Chi P.; Herlyn M.; Holmen S. L.; Karreth F. A.; Kaufman C. K.; Khan S.; Kobold S.; Leucci E.; Levy C.; Lombard D. B.; Lund A. W.; Marie K. L.; Marine J. C.; Marais R.; McMahon M.; Robles-Espinoza C. D.; Ronai Z. A.; Samuels Y.; Soengas M. S.; Villanueva J.; Weeraratna A. T.; White R. M.; Yeh I.; Zhu J.; Zon L. I.; Hurlbert M. S.; Merlino G. Melanoma models for the next generation of therapies. Cancer Cell. 2021, 39 (5), 610–31. 10.1016/j.ccell.2021.01.011. 33545064 PMC8378471

[ref66] Fu C.; Chen J.; Lu J.; Yi L.; Tong X.; Kang L.; Pei S.; Ouyang Y.; Jiang L.; Ding Y.; Zhao X.; Li S.; Yang Y.; Huang J.; Zeng Q. Roles of inflammation factors in melanogenesis (Review). Mol. Med. Rep. 2020, 21 (3), 1421–30. 10.3892/mmr.2020.10950. 32016458 PMC7002987

[ref67] Natarajan V. T.; Ganju P.; Singh A.; Vijayan V.; Kirty K.; Yadav S.; Puntambekar S.; Bajaj S.; Dani P. P.; Kar H. K.; Gadgil C. J.; Natarajan K.; Rani R.; Gokhale R. S. IFN-gamma signaling maintains skin pigmentation homeostasis through regulation of melanosome maturation. Proc. Natl. Acad. Sci. U. S. A. 2014, 111 (6), 2301–6. 10.1073/pnas.1304988111. 24474804 PMC3926048

[ref68] Zhou J.; Ling J.; Wang Y.; Shang J.; Ping F. Cross-talk between interferon-gamma and interleukin-18 in melanogenesis. J. Photochem. Photobiol. B 2016, 163, 133–43. 10.1016/j.jphotobiol.2016.08.024. 27567084

[ref69] Kirkwood J. M.; Lee S.; Moschos S. J.; Albertini M. R.; Michalak J. C.; Sander C.; Whiteside T.; Butterfield L. H.; Weiner L. Immunogenicity and antitumor effects of vaccination with peptide vaccine±granulocyte-monocyte colony-stimulating factor and/or IFN-alpha2b in advanced metastatic melanoma: Eastern Cooperative Oncology Group Phase II Trial E1696. Clin. Cancer Res. 2009, 15 (4), 1443–51. 10.1158/1078-0432.CCR-08-1231. 19228745 PMC2759898

[ref70] Middleton G.; Silcocks P.; Cox T.; Valle J.; Wadsley J.; Propper D.; Coxon F.; Ross P.; Madhusudan S.; Roques T.; Cunningham D.; Falk S.; Wadd N.; Harrison M.; Corrie P.; Iveson T.; Robinson A.; McAdam K.; Eatock M.; Evans J.; Archer C.; Hickish T.; Garcia-Alonso A.; Nicolson M.; Steward W.; Anthoney A.; Greenhalf W.; Shaw V.; Costello E.; Naisbitt D.; Rawcliffe C.; Nanson G.; Neoptolemos J. Gemcitabine and capecitabine with or without telomerase peptide vaccine GV1001 in patients with locally advanced or metastatic pancreatic cancer (TeloVac): an open-label, randomised, phase 3 trial. Lancet Oncol 2014, 15 (8), 829–40. 10.1016/S1470-2045(14)70236-0. 24954781

[ref71] Lawson D. H.; Lee S.; Zhao F.; Tarhini A. A.; Margolin K. A.; Ernstoff M. S.; Atkins M. B.; Cohen G. I.; Whiteside T. L.; Butterfield L. H.; Kirkwood J. M. Randomized, Placebo-Controlled, Phase III Trial of Yeast-Derived Granulocyte-Macrophage Colony-Stimulating Factor (GM-CSF) Versus Peptide Vaccination Versus GM-CSF Plus Peptide Vaccination Versus Placebo in Patients With No Evidence of Disease After Complete Surgical Resection of Locally Advanced and/or Stage IV Melanoma: A Trial of the Eastern Cooperative Oncology Group-American College of Radiology Imaging Network Cancer Research Group (E4697). J. Clin. Oncol. 2015, 33 (34), 4066–76. 10.1200/JCO.2015.62.0500. 26351350 PMC4669592

[ref72] van der Burg S. H.; Arens R.; Ossendorp F.; van Hall T.; Melief C. J. Vaccines for established cancer: overcoming the challenges posed by immune evasion. Nat. Rev. Cancer. 2016, 16 (4), 219–33. 10.1038/nrc.2016.16. 26965076

[ref73] Smith C. C.; Selitsky S. R.; Chai S.; Armistead P. M.; Vincent B. G.; Serody J. S. Alternative tumour-specific antigens. Nat. Rev. Cancer. 2019, 19 (8), 465–78. 10.1038/s41568-019-0162-4. 31278396 PMC6874891

[ref74] Lang F.; Schrors B.; Lower M.; Tureci O.; Sahin U. Identification of neoantigens for individualized therapeutic cancer vaccines. Nat. Rev. Drug Discovery 2022, 21 (4), 261–82. 10.1038/s41573-021-00387-y. 35105974 PMC7612664

[ref75] Sahin U.; Tureci O. Personalized Vaccines for Cancer Immunotherapy. Science. 2018, 359 (6382), 1355–60. 10.1126/science.aar7112. 29567706

[ref76] Lu Z.; Paolella B. R.; Truex N. L.; Loftis A. R.; Liao X.; Rabideau A. E.; Brown M. S.; Busanovich J.; Beroukhim R.; Pentelute B. L. Targeting cancer gene dependencies with anthrax-mediated delivery of peptide nucleic acids. ACS Chem. Biol. 2020, 15 (6), 1358–69. 10.1021/acschembio.9b01027. 32348107 PMC7521945

[ref77] Carvajal R. D.; Butler M. O.; Shoushtari A. N.; Hassel J. C.; Ikeguchi A.; Hernandez-Aya L.; Nathan P.; Hamid O.; Piulats J. M.; Rioth M.; Johnson D. B.; Luke J. J.; Espinosa E.; Leyvraz S.; Collins L.; Goodall H. M.; Ranade K.; Holland C.; Abdullah S. E.; Sacco J. J.; Sato T. Clinical and molecular response to tebentafusp in previously treated patients with metastatic uveal melanoma: a phase 2 trial. Nat. Med. 2022, 28 (11), 2364–73. 10.1038/s41591-022-02015-7. 36229663 PMC9671803

[ref78] Overall Survival Benefit with Tebentafusp in Metastatic Uveal Melanoma. New Engl. J. Med. 2021, 385 (13), 1196–206. 10.1056/NEJMoa2103485. 34551229

