# Supplementary material for: Enhanced Vaccine Immunogenicity Enabled by Targeted Cytosolic Delivery of Tumor Antigens into Dendritic Cells
Source: ACS Cent Sci. 2023 Sep 14;9(9):1835–45. doi: 10.1021/acscentsci.3c00625 (PMC10540291; doi:10.1021/acscentsci.3c00625)
Supplement: Supplementary file 1 — oc3c00625_si_001.pdf [file oc3c00625_si_001.pdf]

Supporting Information for

Enhanced Vaccine Immunogenicity Enabled by Targeted Cytosolic Delivery of Tumor Antigens into Dendritic Cells

Nicholas L. Truex[1,2†], Aurélie Rondon[1†], Simon L. Rössler[1], Cameron C. Hanna[1], Yehlin Cho[3], Bin-You Wang[1], Coralie M. Backlund[4], Emi A. Lutz[4,5], Darrell J. Irvine[3,4,5,6,7]*, and Bradley L. Pentelute[1,4,8,9]*

[1] Department of Chemistry, Massachusetts Institute of Technology, 77 Massachusetts Avenue, Cambridge, Massachusetts 02139, USA.

[2] Department of Chemistry and Biochemistry, University of South Carolina, 631 Sumter Street, Columbia, South Carolina, 29208, USA.

[3] Department of Materials Science and Engineering, Massachusetts Institute of Technology, 77 Massachusetts Avenue, Cambridge, Massachusetts 02139, USA.

[4] The Koch Institute for Integrative Cancer Research, Massachusetts Institute of Technology, 500 Main Street, Cambridge, Massachusetts 02139, USA.

[5] Department of Biological Engineering, Massachusetts Institute of Technology, 77 Massachusetts Avenue, Cambridge, Massachusetts 02139, USA.

[6] Ragon Institute of Massachusetts General Hospital, Massachusetts Institute of Technology and Harvard University, 400 Technology Square, Cambridge, Massachusetts 02139, USA.

[7] Howard Hughes Medical Institute, 4000 Jones Bridge Road, Chevy Chase, Maryland 20815, USA.

[8] Center for Environmental Health Sciences, Massachusetts Institute of Technology, 77 Massachusetts Avenue, Cambridge, Massachusetts 02139, USA.

[9] Broad Institute of MIT and Harvard, 415 Main Street, Cambridge, Massachusetts 02142, USA.

[†] Designates Equal contribution.

* Email: djirvine@mit.edu, blp@mit.edu

I. Supplemental Tables

		page
**Table S1.**	Amino acid and genetic sequence of DC-Targeting scFv.	S4
**Table S2.**	Summary of long antigen peptide sequences.	S5
**Table S3.**	Summary of short epitope peptide sequences.	S5

II. Supplemental Figures

**Fig. S1.**	LC-MS analysis of anti-XCR1 scFv.	S6
**Fig. S2.**	LC-MS analysis of peptides **1a** and **1b**.	S7
**Fig. S3.**	LC-MS analysis of mPAC and mPAC[F427A].	S8
**Fig. S4.**	LC-MS analysis of G_3-mPAC and G_3-mPAC[F427A].	S9
**Fig. S5.**	LC-MS analysis of scFv-mPAC and scFv-mPACA [F427A].	S10
**Fig. S6.**	LC-MS analysis of the AF647-labelled anti-XCR1 scFv.	S11
**Fig. S7.**	Flow cytometry analysis of scFv binding to CHO cells.	S12
**Fig. S8.**	Protein translocation into CHO cells.	S13
**Fig. S9.**	Flow cytometry analysis of scFv binding to murine splenocytes.	S14
**Fig. S10.**	Flow cytometry gating on murine splenocytes (scFv-FAM).	S15
**Fig. S11.**	Flow cytometry gating on murine splenocytes (DTA-FAM).	S16
**Fig. S12.**	LC-MS analysis of the LF_N-$OVA_{252–270}$ conjugate.	S17
**Fig. S13.**	LC-MS analysis of the $OVA_{257–264}$ peptide.	S18
**Fig. S14.**	Flow cytometry analysis of antigen presentation with DC2.4 cells.	S19
**Fig. S15.**	Tetramer and intracellular cytokine staining.	S20
**Fig. S16.**	Intracellular cytokine staining after repeat immunizations.	S21
**Fig. S17.**	LC-MS analysis of the GGG-Trp1-gp100 peptide.	S22
**Fig. S18.**	LC-MS analysis of the LFN-Trp1-gp100 conjugate.	S23
**Fig. S19.**	LC-MS analysis of the $Trp1_{455–463}$ peptide.	S24
**Fig. S20.**	LC-MS analysis of the $gp100_{25–33}$ peptide.	S25
**Fig. S21.**	Phenotype of B16-F10 tumors.	S26

III. MATERIALS AND METHODS

Materials S27
LC-MS analysis S27
Peptide synthesis and purification S28
Development of XCR1-targeting scFv S29
Anthrax protein expression and purification S29
Sortase-mediated ligations S30
Fluorophore ligations S30
Endotoxin testing and removal S31
Cell experiments S31
Cell viability analysis S32
Flow cytometry S32
Animal studies S32
Statistical analysis S34

IV. REFERENCES S35

I. SUPPLEMENTAL TABLES

Table S1. Amino acid sequence and codon optimized gene for the anti-XCR1 scFv.

Description	Fragment
amino acid sequence	QVQLQQPGAELVKPGASVKLSCKASGYTFTNYWIHWMKQRPGQGLEWIGMIHPNSDNTKYNEKFK<br>AKAILTVDKSSSTAYMQLSSLTSEDSAVYYCARFANDGAYWGQGTLVTVSAA**GGGGSGGGGSGG**<br>**GGSGGGGS**DVVVTQTPLSLPVSLGDPASISCKSSQSLVHSNGNTYLHWYLQKPGQSPKLLIYKISNRF<br>SGVPDRFSGSGSGTDFTLKISRVEAEDLGVYFCSQNTHVPYTFGGGTKLEIKR**LPSTGGHHHHHH**
codon optimized gene	CAAGTGCAGCTGCAGCAACCGGGGGCTGAGCTGGTCAAACCGGGCGCTTCCGTCAAACTGTCGT<br>GTAAGGCCAGCGGGTATACTTTCACCAACTATTGGATCCATTGGATGAAACAGCGGCCAGGGCA<br>GGGTTTAGAATGGATTGGTATGATTCATCCAAATTCGGACAATACTAAGTATAACGAGAAATTC<br>AAGGCGAAAGCAATCCTGACGGTCGATAAAAGTAGTAGTACGGCTTACATGCAGCTTTCTAGCC<br>TCACTAGTGAGGATTCCGCTGTGTATTATTGTGCACGGTTCGCAAATGACGGTGCCTACTGGGGG<br>CAGGGGACATTAGTAACTGTTTCTGCAGCT**GGGGGTGGGGGCTCTGGCGGTGGTGGTTCTGG**<br>**GGGCGGTGGGTCTGGTGGCGGCGGTTCT**GATGTAGTCGTAACTCAAACTCCTCTTTCGCTTCC<br>TGTTAGCCTTGGTGATCCGGCTTCCATCAGCTGCAAGAGCTCCCAGTCACTTGTTCATAGCAACG<br>GCAATACATACCTTCACTGGTATCTCCAGAAACCAGGCCAAAGTCCTAAATTGCTTATTTACAAG<br>ATTTCTAACCGGTTCTCTGGGGTACCAGACCGGTTCAGTGGGAGCGGTTCGGGGACAGATTTTAC<br>TCTGAAAATCTCACGTGTGGAGGCGGAAGACTTGGGCGTATATTTCTGCTCTCAGAACACACAC<br>GTTCCTTATACGTTCGGGGGTGGCACCAAGTTGGAGATCAAACGT**CTTCCTTCAACTGGCGGT**<br>**CATCATCACCATCACCAC**

[1] Hydrophilic spacer sequence $(G_4S)_4$ is indicated by bold text with one line underneath, **as shown here**.
[2] Sortase recognition site is indicated by bold text with two lines underneath, **as shown here**.

Table S2. Summary of long antigen peptide sequences, containing N-terminal Gly residues for sortase-mediated ligation to LF_N.

Name	Sequence
GGG-$OVA_{252\text{-}270}$	GGGLEQLESIINFEKLTEWTSS
GGG-$OVA_{257\text{-}264}$	GGGSIINFEKL
GGG-Trp1-gp100	GGGTAPDNLGYMEGPRNQDWL

Table S3. Summary of short epitope peptide sequences.

Name	Sequence
$OVA_{257–264}$	SIINFEKL
$Trp1_{455–463}$	TAPDNLGYM
$Gp100_{25–33}$	EGPRNQDWL

II. SUPPLEMENTAL FIGURES

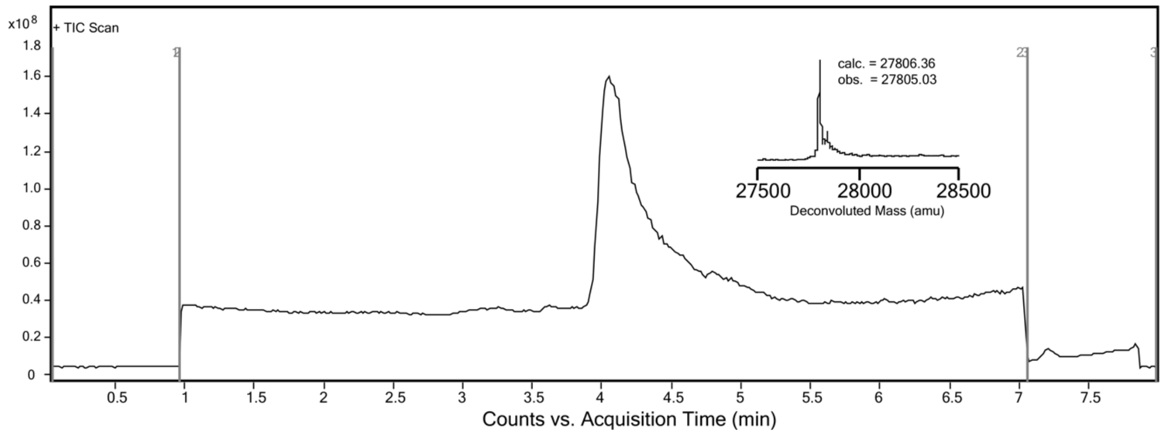

Fig. S1. LC-MS analysis of the anti-XCR1 scFv. The protein (50 ng) was loaded onto an Agilent Zorbax 5 µm 300SB-C3 column (2.1 × 150 mm) and was eluted with a gradient of 1–91% CH_3CN in H_2O with 0.1% FA and a flow rate of 0.5 mL/min. The protein was detected on an Agilent 6550 ESI-Q-TOF mass spectrometer. The TIC peak was integrated and the mass was deconvoluted using maximum entropy algorithm.

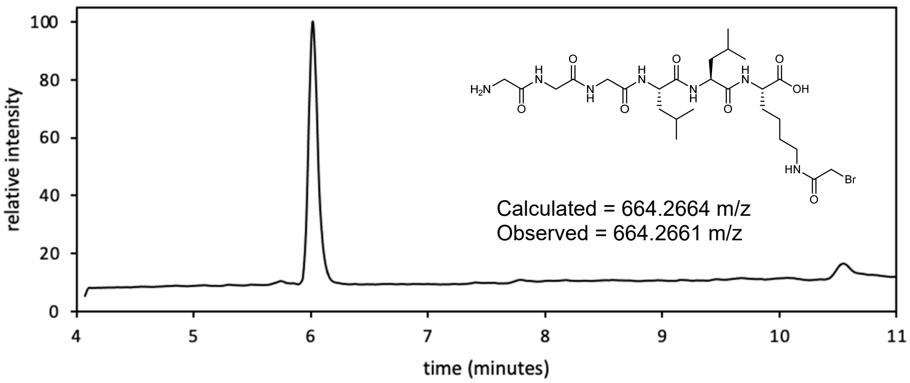

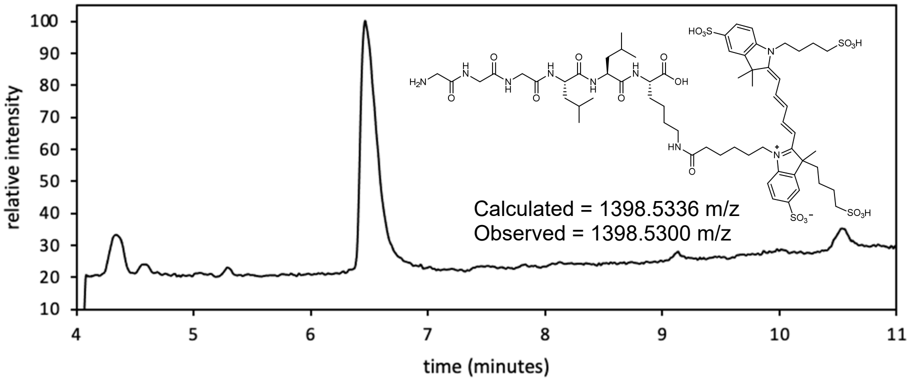

Fig. S2. LC-MS analysis of linker peptides **1a** (top) and **1b** (bottom). The peptides (50 ng) were loaded onto an Agilent Zorbax 5 µm 300SB-C3 column (2.1 × 150 mm) and were eluted with a gradient of 1–91% CH_3CN in H_2O with 0.1% FA and a flow rate of 0.5 mL/min. The peptides were detected on an Agilent 6550 ESI-Q-TOF mass spectrometer. The TIC peak was integrated and the mass was deconvoluted using maximum entropy algorithm.

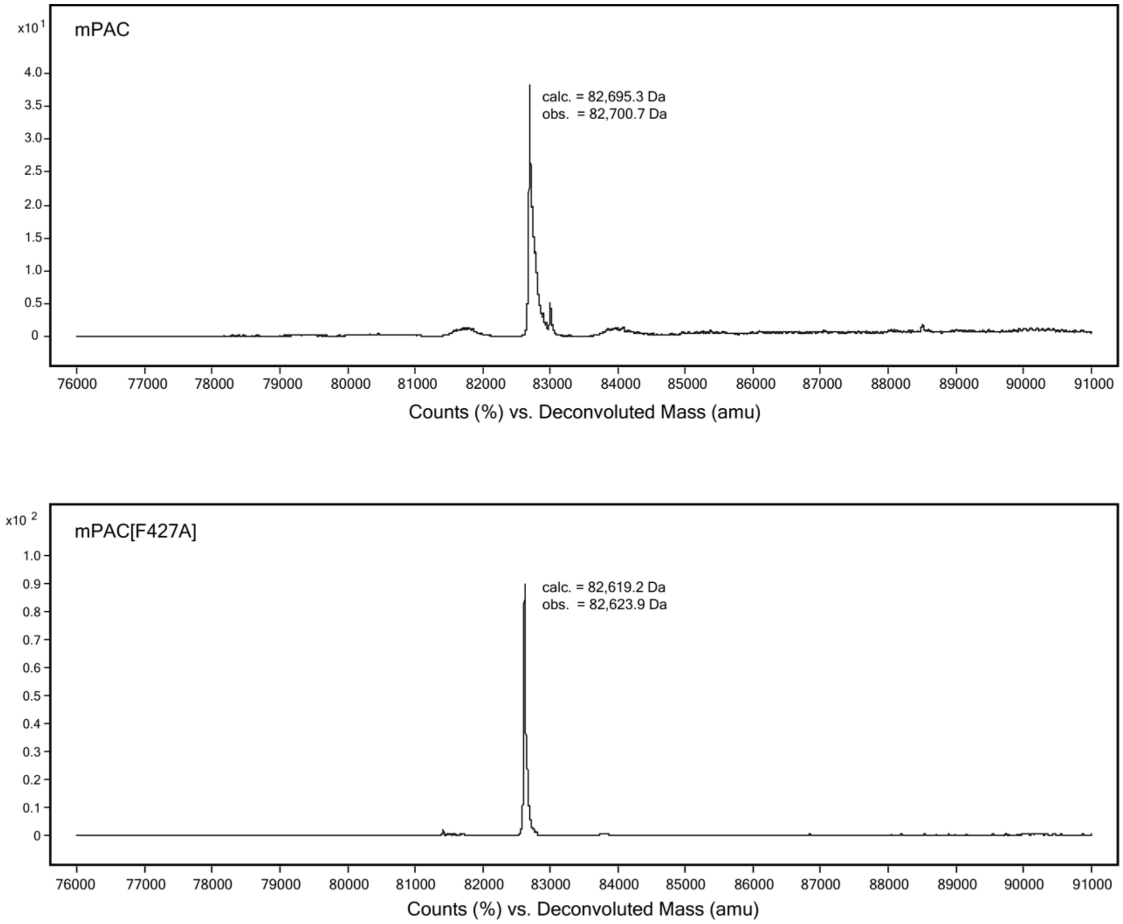

Fig. S3. LC-MS analysis of mPAC and mPAC[F427A]. The proteins (100 ng) were loaded onto an Agilent Zorbax 5 µm 300SB-C3 column (2.1 × 150 mm) and were eluted with a gradient of 1–91% CH_3CN in H_2O with 0.1% FA and a flow rate of 0.5 mL/min. The proteins were detected on an Agilent 6550 ESI-Q-TOF mass spectrometer. The TIC peak was integrated and the mass was deconvoluted using maximum entropy algorithm.

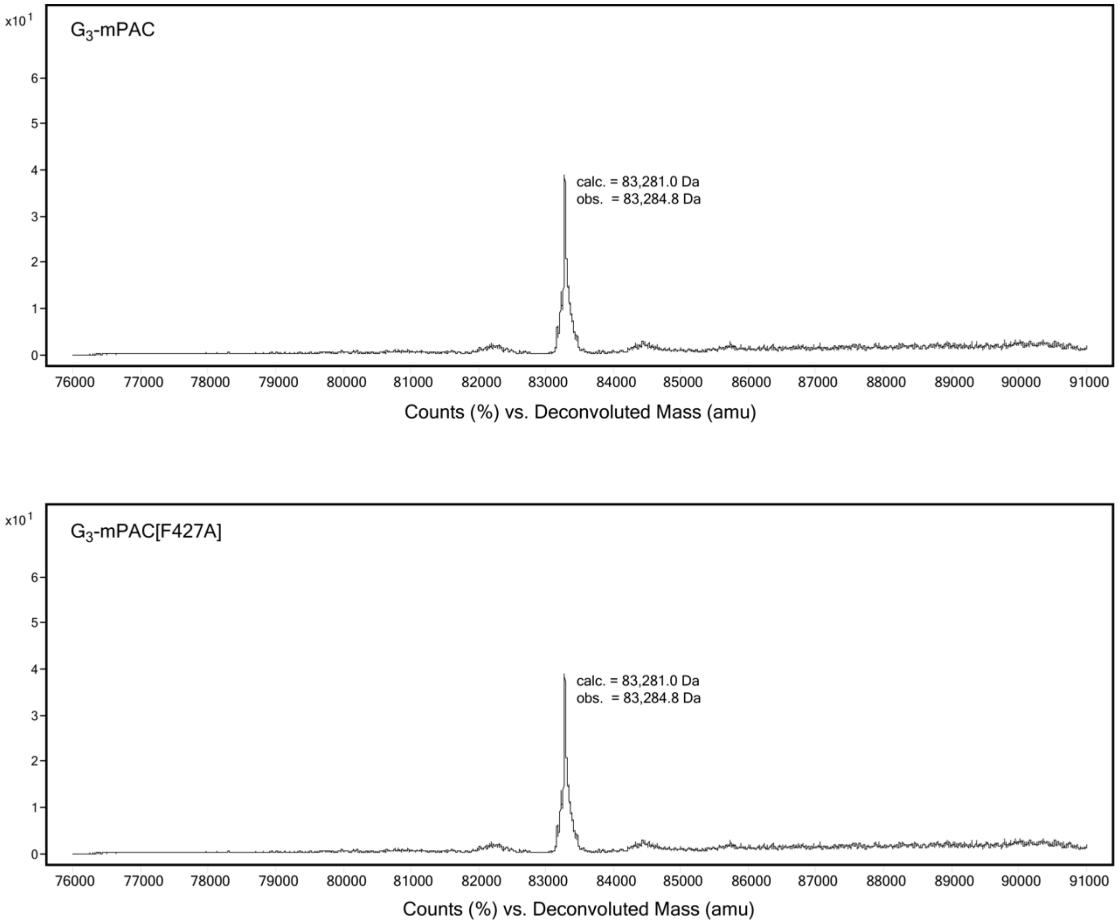

Fig. S4. LC-MS analysis of G_3-mPAC and G_3-mPAC[F427A] after conjugating peptide **1b**. The proteins (100 ng) were loaded onto an Agilent Zorbax 5 μm 300SB-C3 column (2.1 × 150 mm) and were eluted with a gradient of 1–91% CH_3CN in H_2O with 0.1% FA and a flow rate of 0.5 mL/min. The proteins were detected on an Agilent 6550 ESI-Q-TOF mass spectrometer. The TIC peak was integrated and the mass was deconvoluted using maximum entropy algorithm.

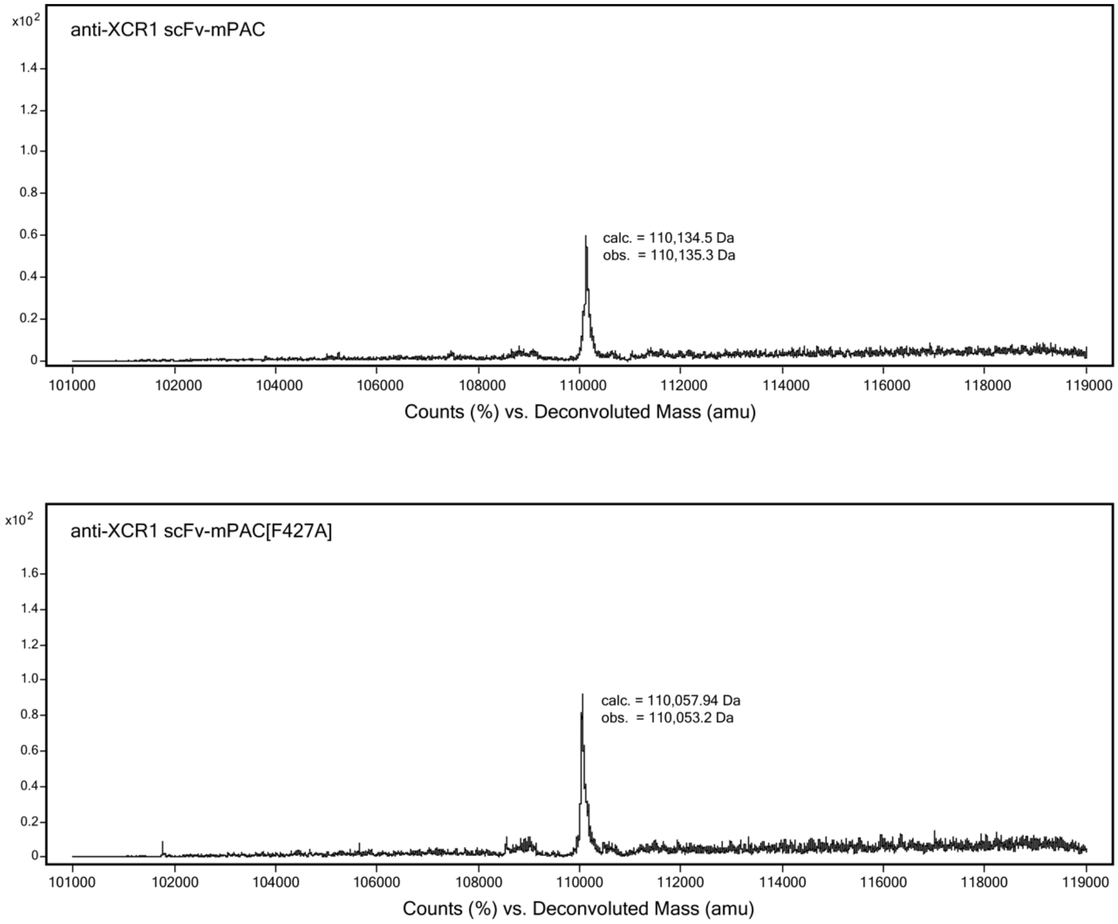

Fig. S5. LC-MS analysis of scFv-mPAC and scFv-mPAC[F427A]. The protein (100 ng) was loaded onto an Agilent Zorbax 5 μm 300SB-C3 column (2.1 × 150 mm) and were eluted with a gradient of 1–91% CH_3CN in H_2O with 0.1% FA and a flow rate of 0.5 mL/min. The proteins were detected on an Agilent 6550 ESI-Q-TOF mass spectrometer. The TIC peak was integrated and the mass was deconvoluted using maximum entropy algorithm.

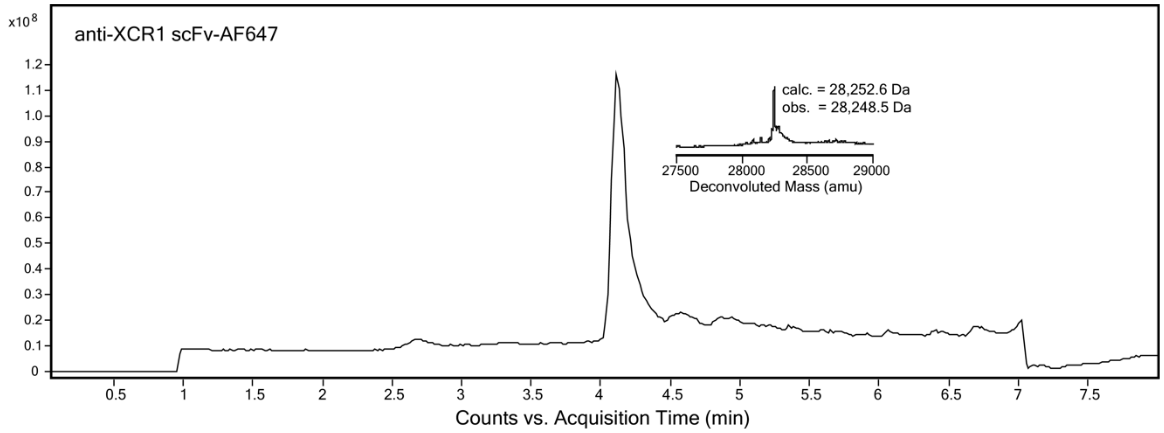

Fig. S6. LC-MS analysis of the AF647-labelled anti-XCR1 scFv after conjugating peptide **1a**. The protein (50 ng) was loaded onto an Agilent Zorbax 5 µm 300SB-C3 column (2.1 × 150 mm) and was eluted with a gradient of 1–91% CH_3CN in H_2O with 0.1% FA and a flow rate of 0.5 mL/min. The protein was detected on an Agilent 6550 ESI-Q-TOF mass spectrometer. The TIC peak was integrated and the mass was deconvoluted using maximum entropy algorithm.

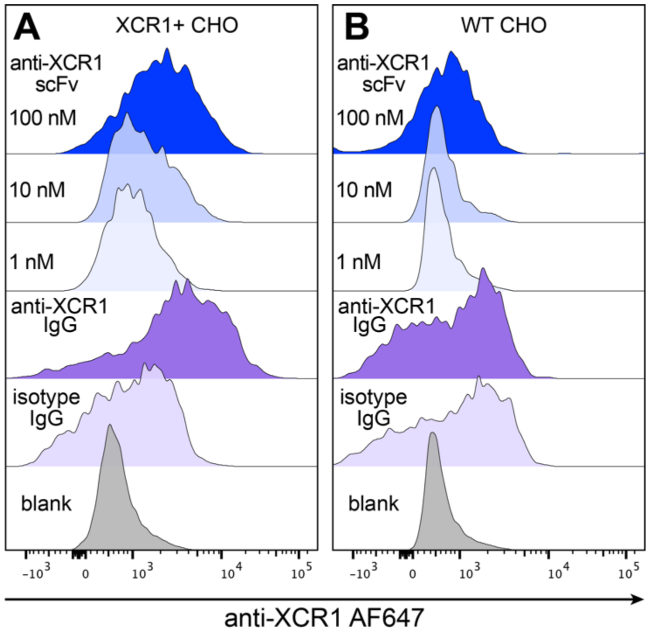

Fig. S7. Flow cytometry analysis of scFv binding to CHO cells. Flow cytometry analysis of scFv binding to two CHO cell lines: (A) $XCR1^{+}$ and (B) $XCR1^{-}$. Representative plots showing binding to cells after 30 min incubation with AF647-labelled anti-XCR1 scFv, anti-XCR1 IgG, and isotype IgG constructs.

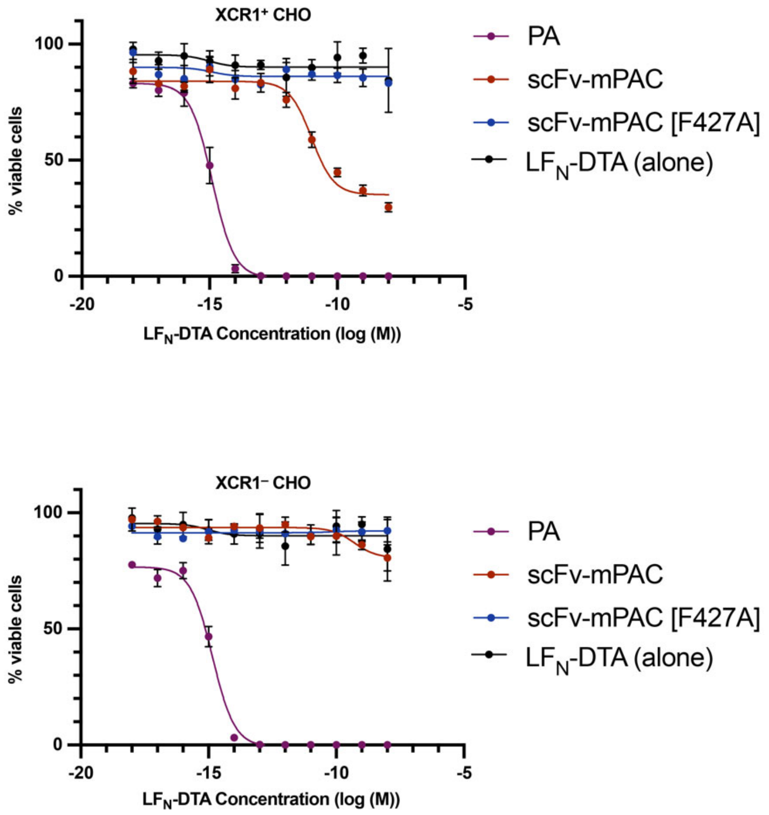

Fig. S8. Protein translocation into CHO cells. Relative cell viability from translocated LF_N-DTA into (top) $XCR1^+$ and (bottom) $XCR1^-$ CHO cells. Cells were incubated (72 h) with ten-fold serial dilutions of LF_N-DTA in the presence of 20 nM PA, scFv-mPAC, or scFv-mPAC[F427A]. Relative viability (% viable cells) was determined from a Cell Titer-Glo assay; viability was normalized to untreated cells. Data represent the mean of three replicate wells ± the standard deviation (s.d.). Data are representative of two independent experiments.

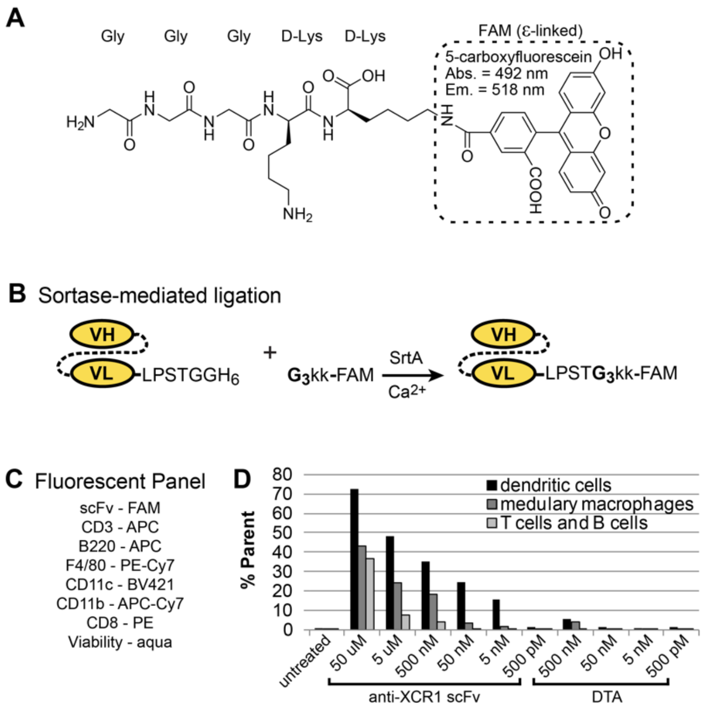

Fig. S9. Flow cytometry analysis of scFv binding to murine splenocytes. (A) 5-carboxyfluorescein (FAM) with a G_3 linker peptide. (B) Sortase-mediated ligation of the anti-XCR1 scFv and FAM. (C) Fluorescent panel for identifying cross-presenting dendritic cells in murine splenocytes by flow cytometry. (G) Flow cytometry results showing binding to DCs, medullary macrophages, and T cells and B cells from murine splenocytes treated with FAM-labeled scFv and a diphtheria toxin protein (DTA, negative control). Data are representative of two independent experiments.

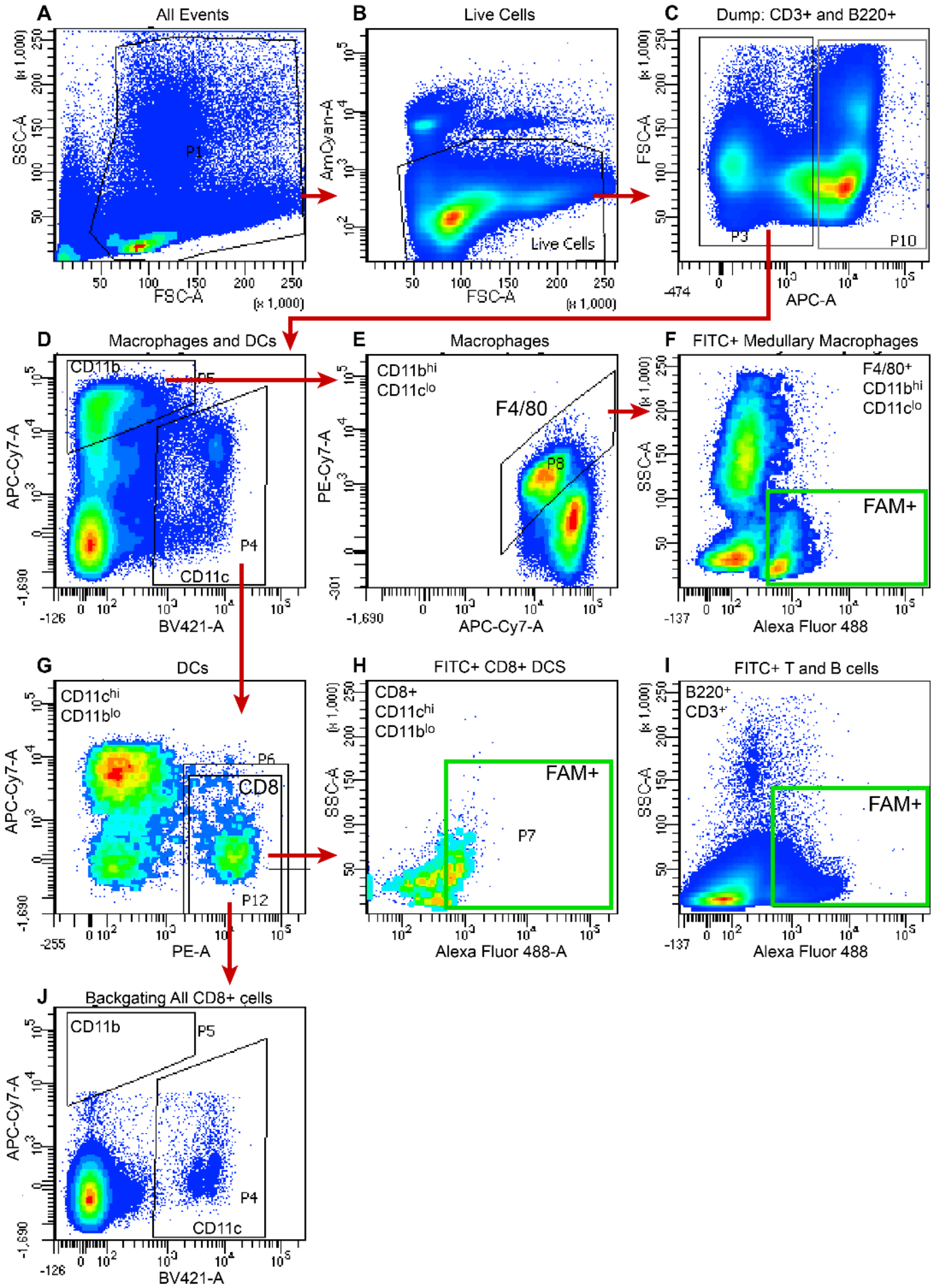

Fig. S10. Flow cytometry gating on murine splenocytes (scFv-FAM). (A-J) Flow cytometry analysis of murine splenocyte populations after 30 min incubation with FAM-labeled scFv. (F, H, I) Analysis of FAM-positive populations. Data are representative of two independent experiments.

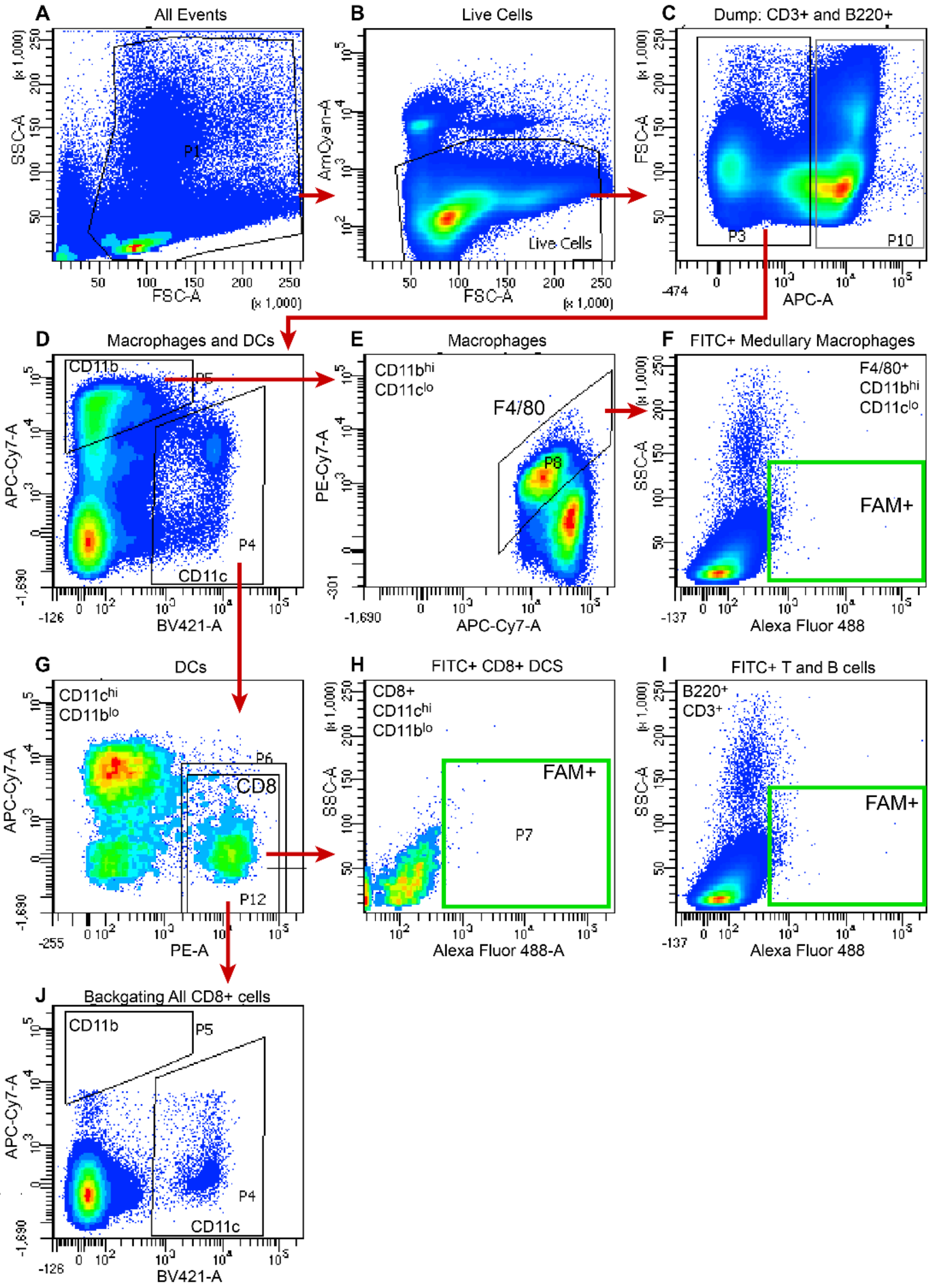

Fig. S11. Flow cytometry gating on murine splenocytes (DTA-FAM). (A-J) Flow cytometry analysis of murine splenocyte populations after 30 min incubation with FAM-labeled DTA (negative control). (F, H, I) Analysis of FAM-positive populations. Data are representative of two independent experiments.

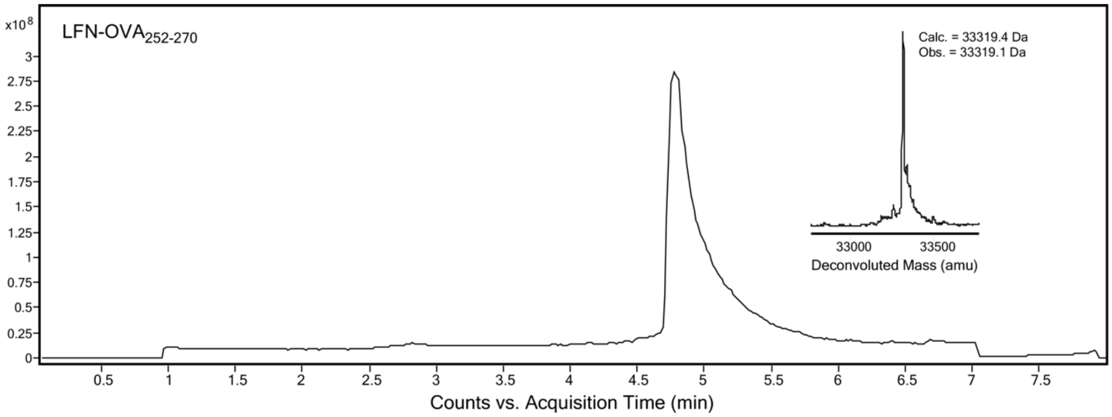

Fig. S12. LC-MS analysis of the LF_N-$OVA_{252–270}$ (GGGLEQLESIINFEKLTEWTSS) conjugate. The protein (50 ng) was loaded onto an Agilent Zorbax 5 μm 300SB-C3 column (2.1 × 150 mm) and was eluted with a gradient of 1–91% CH_3CN in H_2O with 0.1% FA and a flow rate of 0.5 mL/min. The protein was detected on an Agilent 6550 ESI-Q-TOF mass spectrometer. The TIC peak was integrated and the mass was deconvoluted using maximum entropy algorithm.

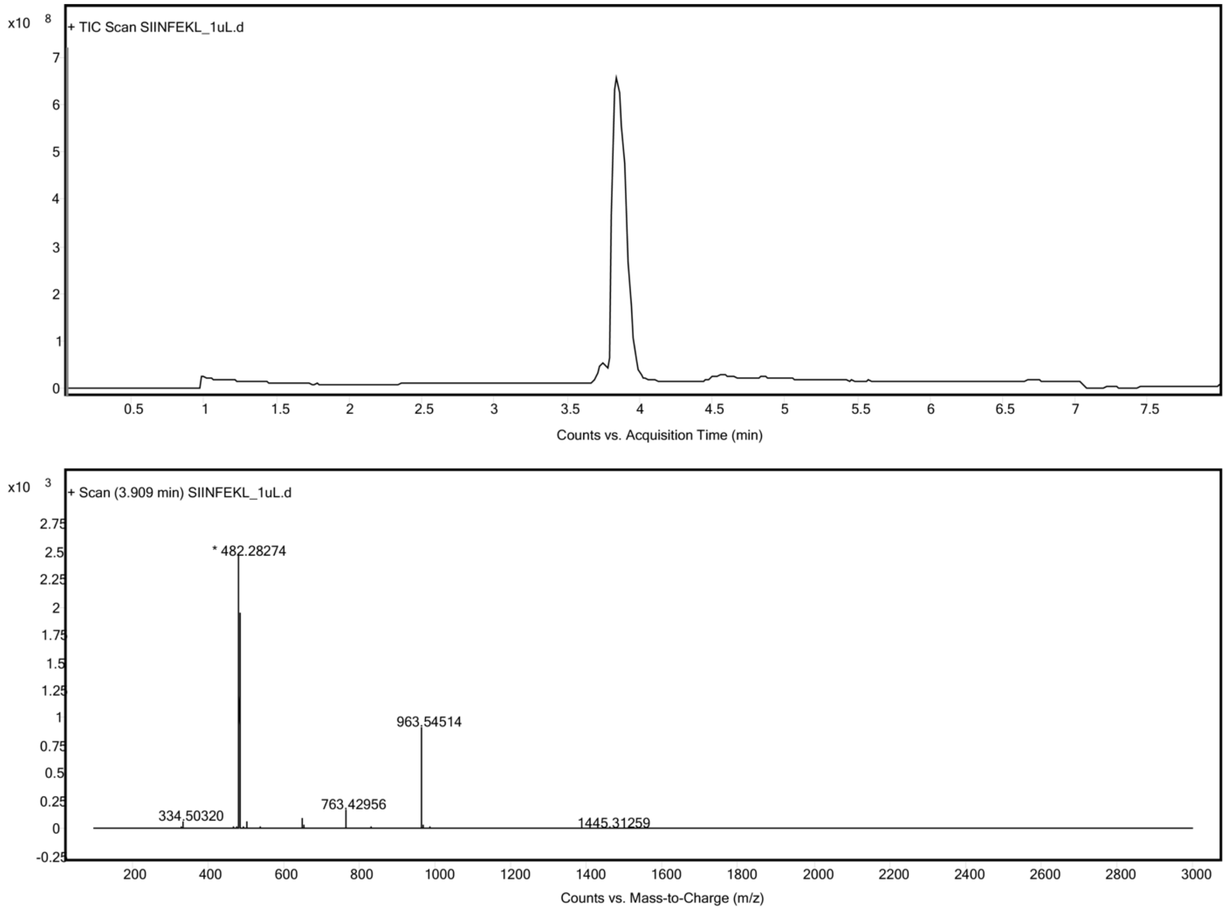

Fig. S13. LC-MS analysis of the $OVA_{257–264}$ (SIINFEKL) peptide. The peptide (10 ng) was loaded onto an Agilent Zorbax 5 µm 300SB-C3 column (2.1 × 150 mm) and was eluted with a gradient of 1–91% CH_3CN in H_2O with 0.1% FA and a flow rate of 0.5 mL/min. The peptide was detected on an Agilent 6550 ESI-Q-TOF mass spectrometer.

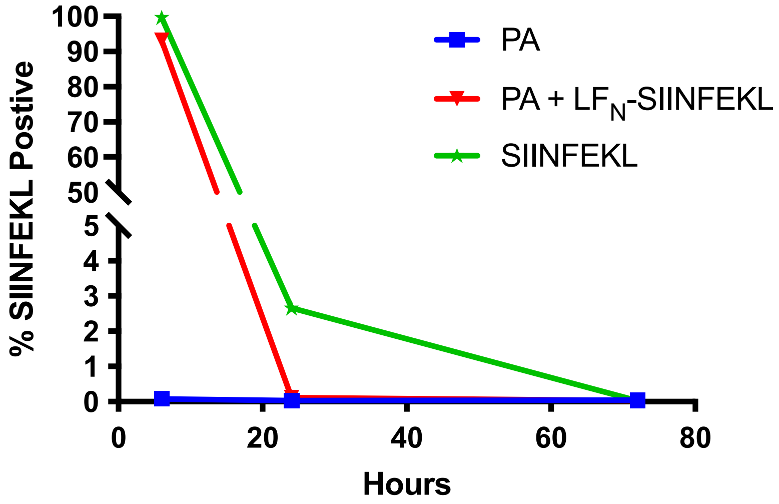

Fig. S14. Flow cytometry analysis of antigen presentation with DC2.4 cells. Flow cytometry analysis with DC 2.4 cells of MHC class I molecule Kb bound to the peptide SIINFEKL. Data was obtained at 6, 24, and 72 h post-treatment with either 20 nM PA, 20 nM PA + 1 µM LF_N-SIINFEKL, or 1 µM SIINFEKL.

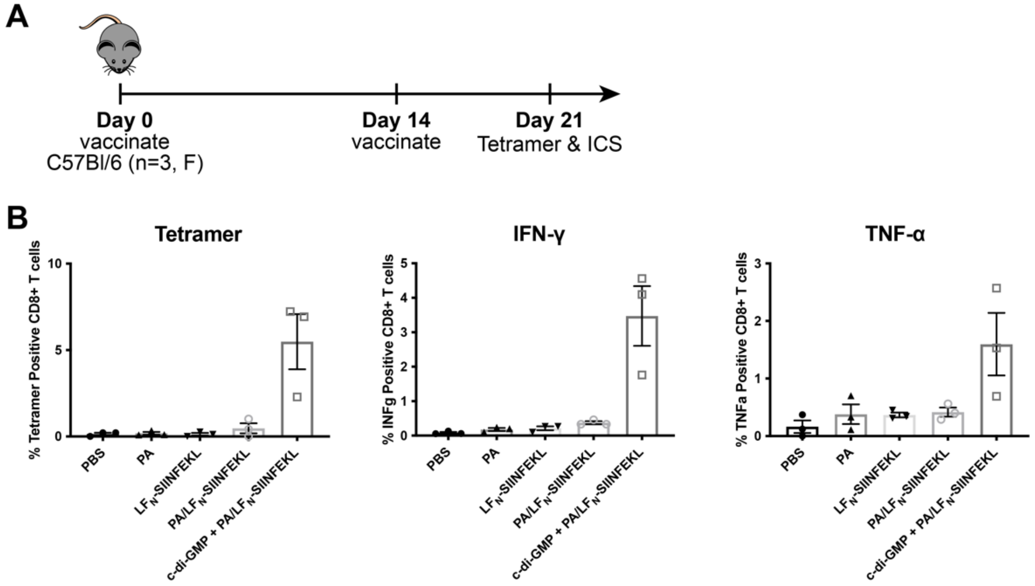

Fig. S15. Tetramer and intracellular cytokine staining. (A) On days 0 and 14, the mice were subcutaneously (s.c.) vaccinated with PA (6 pmol), LF_N-$OVA_{257–264}$ (30 pmol), and c-di-GMP (25 μg). On day 21, antigen-specific immunity was evaluated on peripheral blood cells after ex vivo stimulation with $OVA_{257–264}$ (Table S3) followed by tetramer and intracellular cytokine (IFN-γ and TNF-α) staining. (B) Tetramer and intracellular cytokine staining plots. Data represent the mean ± SEM (n = 3 mice per group).

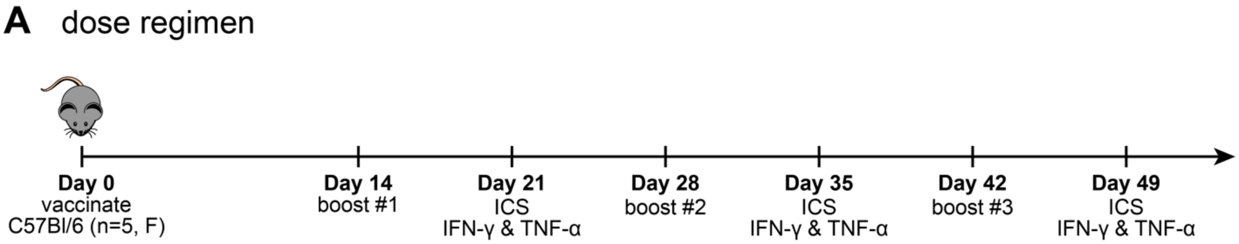

B control groups

	PA (pmol)	LF_N-SIINFEKL (pmol)	c-di-GMP (µg)	vehicle
1	–	–	–	PBS
2	6	30	25	PBS
3	6	300	25	PBS
4	6	600	25	PBS
5	60	30	25	PBS
6	60	300	25	PBS
7	60	600	25	PBS

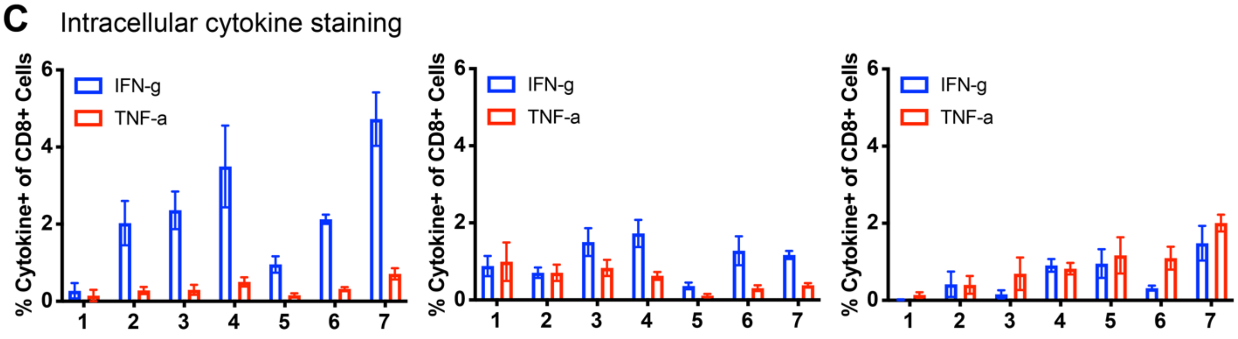

Fig. S16. Intracellular cytokine staining after repeat immunizations. (A) On days 0, 14, 28, and 42, mice were s.c. vaccinated with PA, LF_N-OVA ($OVA_{257–264}$), and c-di-GMP (25 µg). Seven days after each boost, ex vivo stimulation with $OVA_{257–264}$ (Table S3) and intracellular cytokine staining (ICS) was used to evaluate levels of IFN-γ and TNF-α. (B) Summary of control groups. (C) Intracellular cytokine staining of IFN-γ and TNF-α are shown after each boost. Data represent the mean ± SEM (n = 5 mice per group).

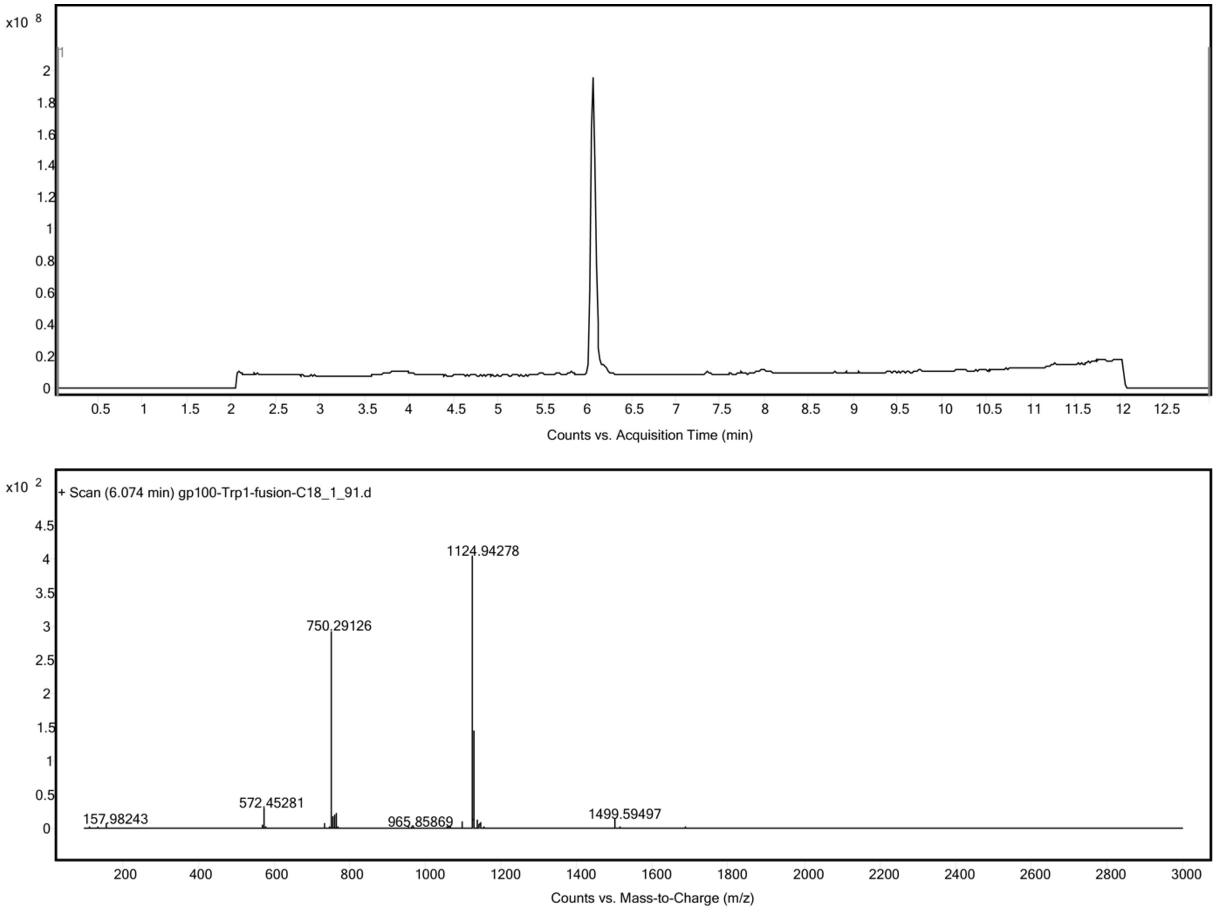

Fig. S17. LC-MS analysis of GGG-Trp1-gp100 (GGGTAPDNLGYMEGPRNQDWL). The peptide (10 ng) was loaded onto an Agilent Zorbax 5 µm 300SB-C3 column (2.1 × 150 mm) and was eluted with a gradient of 1–91% CH_3CN in H_2O with 0.1% FA and a flow rate of 0.5 mL/min. The peptide was detected on an Agilent 6550 ESI-Q-TOF mass spectrometer.

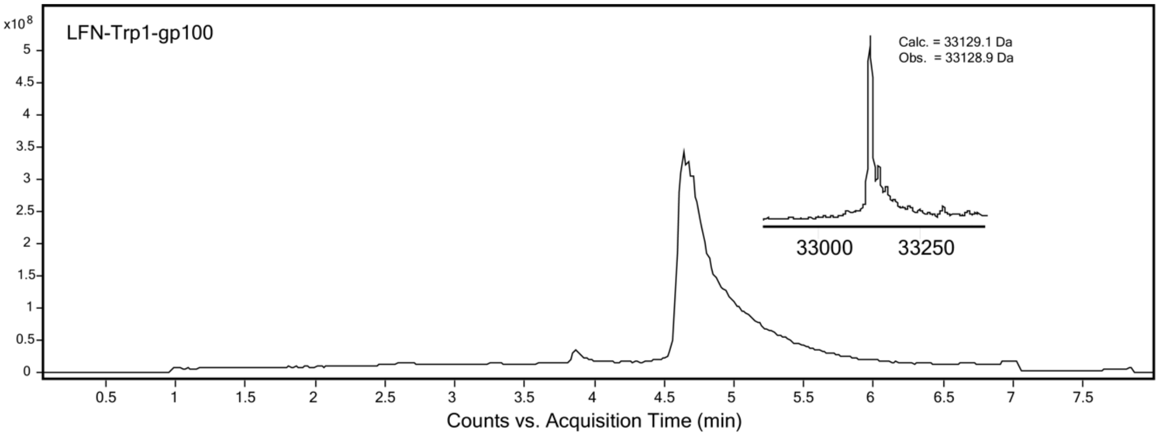

Fig. S18. LC-MS analysis of LF_N-Trp1-gp100 conjugate. The protein (50 ng) was loaded onto an Agilent Zorbax 5 μm 300SB-C3 column (2.1 × 150 mm) and was eluted with a gradient of 1–91% CH_3CN in H_2O with 0.1% FA and a flow rate of 0.5 mL/min. The protein was detected on an Agilent 6550 ESI-Q-TOF mass spectrometer. The TIC peak was integrated and the mass was deconvoluted using maximum entropy algorithm.

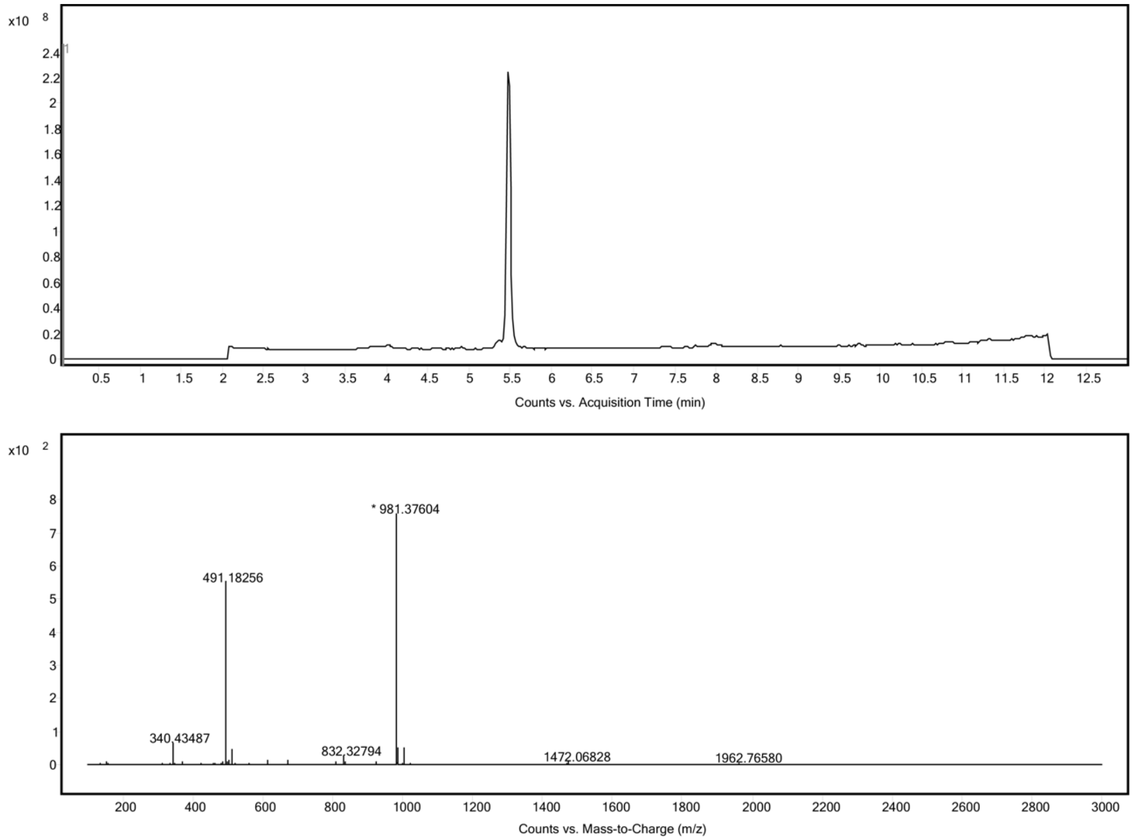

Fig. S19. LC-MS analysis of the Trp1$_{455–463}$ (TAPDNLGYM) peptide. The peptide (10 ng) was loaded onto an Agilent Zorbax 5 µm 300SB-C3 column (2.1 × 150 mm) and was eluted with a gradient of 1–91% CH_3CN in H_2O with 0.1% FA and a flow rate of 0.5 mL/min. The peptide was detected on an Agilent 6550 ESI-Q-TOF mass spectrometer.

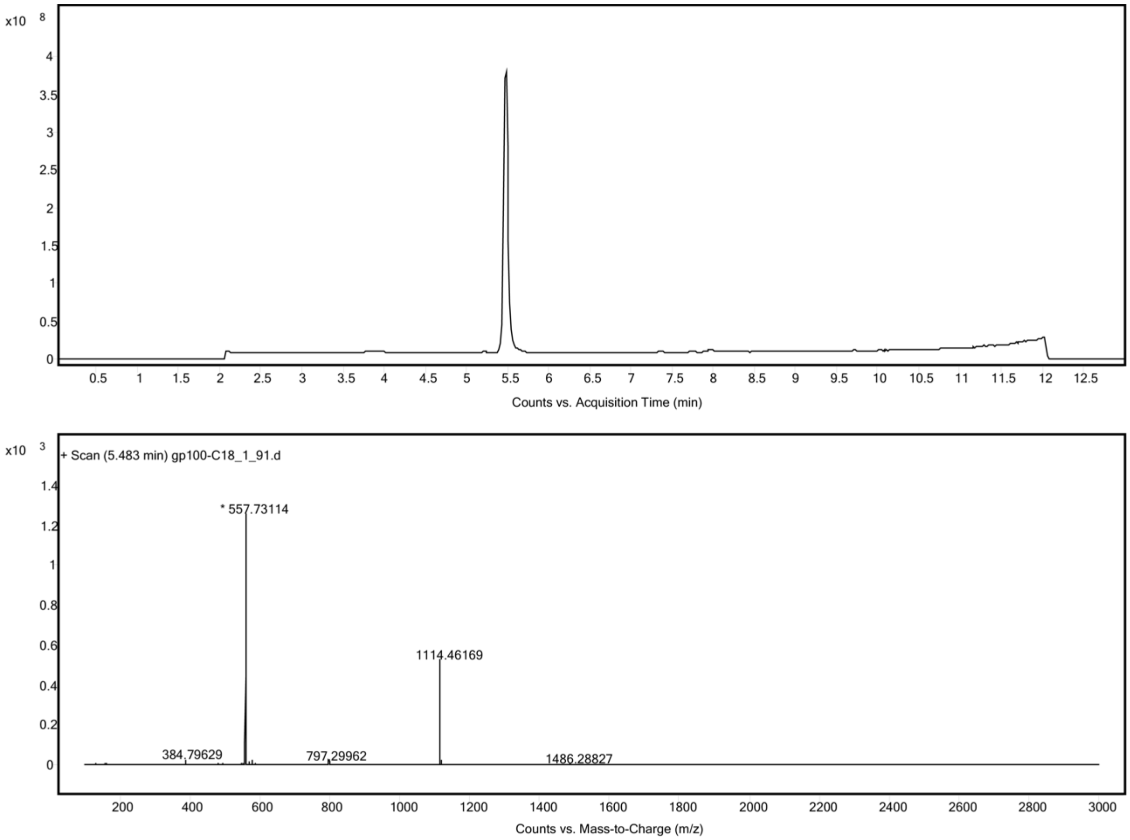

Fig. S20. LC-MS analysis of gp100$_{25–33}$ (EGPRNQDWL). The peptide (10 ng) was loaded onto an Agilent Zorbax 5 μm 300SB-C3 column (2.1 × 150 mm) and was eluted with a gradient of 1–91% CH_3CN in H_2O with 0.1% FA and a flow rate of 0.5 mL/min. The peptide was detected on an Agilent 6550 ESI-Q-TOF mass spectrometer.

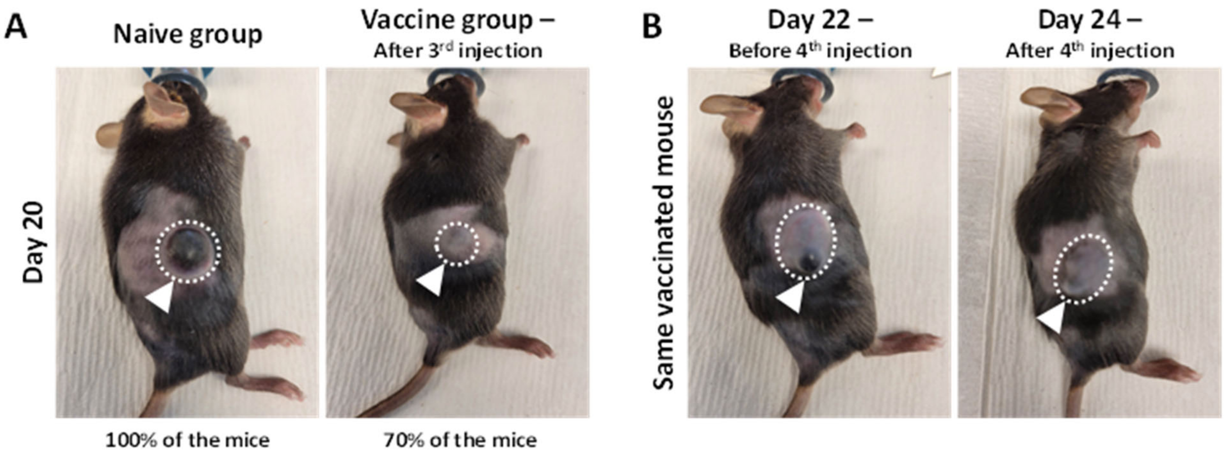

Fig. S21. Phenotype of B16-F10 tumors. (A) Comparison between the control mice (naive group) and the mice in the vaccine group (LF_N-Trp1-gp100/ ScFv-mPAC), after the 3rd vaccine injection. (B) Observation of a representative mouse, among the 30% of mice from the vaccine group (LF_N-Trp1-gp100/ ScFv-mPAC) with partially pigmented tumors, at Day 22 (before the 4th vaccine injection) and Day 24 (after the 4th vaccine injection). The white arrows indicate the localization of the tumor, the white circle indicates its circumference.

III. MATERIALS AND METHODS

Materials. Fmoc-protected L-amino acids used for peptide synthesis were purchased from Novabiochem. Peptide synthesis couplings were performed with 1-[bis-(dimethylamino)methylene]-1H-1,2,3-triazolo[4,5-b]-pyridinium 3-oxid hexafluorophosphate (HATU) and (7-Azabenzotriazol-1-yloxy)tripyrrolidinophosphonium hexafluorophosphate (PyAOP), which were purchased from P3 Biosystems. Dimethylformamide, piperidine, diisopropylethylamine, trifluoroacetic acid, and triisopropylsilane were purchased from VWR or Sigma-Aldrich. Antibodies for flow cytometry were purchased from BioLegend. Media for cell culture were purchased from ThermoFisher Scientific. Tissue culture was performed with RPMI 1640 Medium, GlutaMAX™ Supplement, Fetal Bovine Serum, qualified, One Shot™ format. Penicillin-Streptomycin (10,000 U/mL) was purchased from ThermoFisher Scientific. DMEM-F12K medium was purchased from ATCC.

LC-MS analysis. Performed on an Agilent 6550 ESI-Q-TOF mass spectrometer, equipped with an Agilent 1260 LC stack. RP-HPLC was performed with a Zorbax 300SB-C3 column (2.1 mm × 150 mm, 5 μM) using a 1–91% gradient of CH_3CN in H_2O with 0.1% formic acid and a flow rate of 0.5 mL/min.

Peptide Synthesis and Purification.

Antigen peptides. These peptides (Tables S2 and S3) were synthesized on a 0.1 mmol scale by automated flow peptide synthesis, according to previously-established procedures (*1*). The syntheses were performed on ChemMatrix resin with a 4-(4-Hydroxymethyl-3-methoxyphenoxy)butyric acid (HMPB) linker (200 mg, 0.5 mmol/g, 100–200 mesh). The first amino acid (1 mmol, 10 equiv.) was manually coupled to the resin with DIC (0.5 mmol, 78 μL) and DMAP (0.01 mmol, 50 μL of a 0.2M solution in DMF) in 3.17 mL of DMF. The resin suspension was incubated overnight (16–24 h), then was drained and rinsed three times with DMF (5 mL). Subsequent amino acids were added by automated flow peptide synthesis.(*1-3*) After the syntheses were complete, peptide cleavage and global deprotection was performed with a solution of trifluoroacetic acid, water, ethane dithiol, and triisopropyl silane (94/2.5/2.5/1). Purification was achieved by preparative RP-HPLC with an Agilent Zorbax SB-C18 Prep HT column (21.2 mm × 250 mm, 7 μm) at a flow rate of 15 mL/min using a gradient with water and acetonitrile containing 0.1% TFA. Pure HPLC fractions were pooled and lyophilized. The purified peptides were analyzed by LC-MS.

*Linker peptides **1***. These peptides were manually synthesized by solid phase peptide synthesis. To a solution of Fmoc-Lys(alloc)-OH (7.0 equiv) in DMF (0.4 M) was added DIC (5.0 equiv). After 2 minutes, the solution was added to HMPB ChemMatrix resin (0.44 mmol/g loading, 1.0 equiv), followed by DMAP (0.2 M in DMF, 0.10 equiv). The resin was gently agitated for 14 hours, followed by Fmoc deprotection (20% piperidine in DMF, 2 x 5 min). Subsequently, Fmoc-D-Leu-OH (2x), then Fmoc-Gly-OH (2x), and lastly Boc-Gly-OH were coupled using amino acid (5.0 equiv) in HATU (0.38 M in DMF, 4.5 equiv) with DIPEA (15 equiv) for 15 minutes, followed by Fmoc deprotection (20% piperidine in DMF, 2 x 5 min). The Alloc group was removed using tetrakis(triphenylphosphine)palladium (0.1 equiv) and phenylsilane (10 equiv) in DCM twice for 30 minutes. The resin was washed using sodium diethyldithiocarbamate (0.5% in DMF, 3 x 2 min) to remove residual palladium.

- For peptide **1a**, bromoacetic acid was coupled to the side chain of lysine using standard SPPS conditions as specified above.
- For peptide **1b**, AZDye 647 dye was coupled to the side chain of lysine by through gentle agitation of the resin, AZDye 647 NHS Ester (0.9 equiv) and DIPEA (1 equiv) in DMF for 16 h.

The peptides were cleaved with a solution of TFA/H2O/TIPS (95:2.5:2.5) and purified by automated column chromatography (C18, 5 to 40% MeCN in H2O with 0.1% TFA). Pure HPLC fractions were pooled, lyophilized, and analyzed by LC-MS.

Development of XCR1-targeting scFv.

Plasmid construction. The scFv plasmid was constructed from an anti-XCR1 IgG (clone: MARX10), comprising amino acid sequence from the IgG variable heavy (VH) and light (VL) chains, a hydrophilic spacer region $(G_4S)_4$ between the two chains, and a sortase recognition tag (LPSTGG) at the C-terminus. The codon-optimized gene (Integrated DNA Technologies) encoding the scFv was cloned into a pET-SUMO vector (Invitrogen), according to the manufacturer's instructions. The mixture was transformed into Mach1™ T1 phage-resistant *E. coli,* streaked on an LB Agar plate (30 µg/mL Kanamycin), and incubated for 12 h at 37 ºC. Individual colonies were selected and expanded in 10-mL cultures of LB Broth containing 30 µg/mL Kanamycin. After incubating for 12 h at 37 ºC and 180 rpm, plasmids were recovered using a miniprep kit (QIAGEN) according to the manufacturer's instructions. Incorporation of the gene insert was established using Sanger sequencing.

Recombinant expression. The scFv protein was expressed as a SUMO fusion protein with SHuffle® T7 Express Competent *E. coli* (New England Biolabs). The plasmid was transformed into *E. coli*, streaked onto an LB Agar plate (30 µg/mL Kanamycin), and incubated at 30 ºC. After 15–24 h, individual colonies were selected, expanded into larger cultures with Terrific Broth (0.4% v/v glycerol, 30 µg/mL Kanamycin), and incubated at 30 ºC and 180 rpm. After OD600 reached ~0.6–0.8, protein expression was induced with 0.4 mM isopropyl β- d-1-thiogalactopyranoside (IPTG) and incubated at 18 ºC and 130 rpm. After 24 h, the cultures were harvested by centrifugation (10,000 × g for 10 min.) and stored at -80 ºC.

Purification. The recombinant SUMO-scFv fusion protein was obtained by suspending the bacteria pellet in Tris-buffered saline (20 mM Tris, pH 8.5, 150 mM NaCl), containing cOmplete™ Protease Inhibitor Cocktail (Roche). The mixture was lysed by sonication and centrifuged (10,000 × g for 30 min.). The supernatant was loaded onto a 5-mL HisTrap FF Ni-NTA column (Cytiva), then eluted with imidazole-buffered saline (0.5 M Im., 20 mM Tris, pH 8.5, 150 mM NaCl). Fractions containing the SUMO-scFv were combined, concentrated, loaded onto a size-exclusion chromatography column (SEC, HiLoad 16/600 Superdex 200), and eluted with Tris-buffered saline (20 mM Tris, pH 7.5, 150 mM NaCl). Clean fractions were combined, concentrated (10 mL), and treated with SUMO protease. After incubating for 24 h at 4 ºC, buffer was added for three-fold dilution to achieve a reduced-saline Tris buffer (20 mM Tris, pH 7.5, 50 mM NaCl). The mixture was loaded onto a 5-mL HiTrap Q HP anion exchange chromatography column, then the immediate flow-through solution gave the purified scFv, without the SUMO domain. The solution was collected, concentrated (ca. 5 mg/mL), flash frozen in liquid nitrogen, and stored at -80 ºC. This workflow typically afforded 10 mg/L of the anti-XCR1 scFv.

Anthrax protein expression and purification.

Protective antigen. PA, mPAC, and mPAC[F427A] variants were expressed in BL21(DE3) *E. coli* (New England Biolabs) at the New England Regional Center of Excellence/Biodefense and Emerging Infectious Diseases (NERCE), as described previously (*4*). After the expression, PA was isolated from the *E. coli* pellets through osmotic shock by suspending the cells in a sucrose buffer (20 mM Tris pH 8.5, 1 mM EDTA, 20% sucrose), followed by pelleting and resuspending the cells in a 5 mM MgSO4 buffer. PA was purified by pelleting the cell lysate, filtering the supernatant through a 0.2 µm filter, and purifying the filtrant by anion exchange chromatography.

N-terminus of Lethal factor (LF_N). LF_N and LF_N-DTA variants were expressed as SUMO fusion proteins in a Champion pET-SUMO vector: SUMO-LF_N-LPSTGG-H_6 and SUMO-LF_N-DTA. After expression, the proteins were isolated from *E. coli* pellets by suspension in Tris buffer (20 mM Tris, 150 mM NaCl, pH 8.5), lysis by sonication, and purification with a HisTrap FF Ni-NTA column. The proteins were eluted with Tris buffer containing 0.5 M imidazole, then buffer exchanged to remove imidazole. SUMO was cleaved using SUMO protease. Afterward, LF_N was used without further purification for protein ligation reactions with SrtA*. LF_N-DTA was further purified by size-exclusion chromatography. Purified LF_N and LF_N-DTA were analyzed by SDS-PAGE at 165 V for 36 min on an Invitrogen Bolt™ 4–12% Bis-Tris Plus Gel with Bolt™ MES SDS Running Buffer (1x). Gels were visualized by SimplyBlue™ SafeStain (Coomassie). Clean fractions were pooled, concentrated with an Amicon® Ultra-15 Centrifugal Filter Unit, and analyzed by LC-MS.

Sortase-mediated ligations.

PA Sortase Ligations. scFv-mPAC and scFv-mPAC[F427A] were prepared by enzymatic ligation using SrtA*, according to previously-developed conditions (*5*). mPAC (400 μM) was incubated with linker peptide **1b**, (2 mM) in 20 mM Tris and 150 mM NaCl (pH 8.5). After 1 h, the excess peptide was removed by three rounds of buffer exchange with a 30 kDa Amicon ultra-15 centrifugal filter (Millipore). The resulting G_3-mPAC (100 μM) was incubated with scFv-LPSTGG-H_6 (100 μM) in the presence of 10 μM of triple mutant sortase (SrtA*) in Tris buffer (20 mM Tris, pH 7.5, 150 mM NaCl) containing 10 mM $CaCl_2$. After 1 h, the reaction mixture was loaded onto a HiLoad 16/600 Superdex 200 pg size exclusion column (GE). The fractions were analyzed by SDS-PAGE. Fractions containing the scFv-mPAC were pooled, concentrated, and buffer exchanged into Tris buffer (20 mM Tris, 50 mM NaCl, pH 8.5) for anion exchange chromatography. The scFv-mPAC was loaded onto a 5-mL HiTrap Q column (GE), then eluted with Tris buffer (20 mM Tris, pH 8.5) containing 0.5 M NaCl. The molecular weight for the purified scFv-mPAC (or scFv-mPAC[F427A]) was confirmed by LC-MS.

LF_N Sortase Ligations. Antigenic peptides (Table S2) were conjugated to the C-terminus of LF_N using enzymatic ligation with SrtA*, according to previously-developed conditions (*6*). LF_N-LPSTGG-H_6 (80 μM) was combined with the GGG-peptides (800 μM), SrtA* (10 μM), and 0.5 M $CaCl_2$ (for a final buffer concentration of 10 mM $CaCl_2$). The reaction mixture was gently rotated for 45 min., followed by adding triple-rinsed Ni-NTA agarose beads (50 μL per mg of protein to isolate: H_6-SrtA*; reacted starting material, GG-H_6; and unreacted starting material, LF_N-LPSTGG-H_6). The bead mixture was centrifuged (30 sec × 16,000 rpm) to collect the reaction supernatant, followed successive rounds of rinsing with PBS at pH 8.5 (3 × 0.5 mL). The rinses were combined and pushed through a 0.2 um syringe filter. To the mixture was added aqueous EDTA (0.5 M, 100 μL) for sequestering the $CaCl_2$. To remove the remaining peptide, the mixture was buffer exchanged with four rounds of Amicon® Ultra-30 Centrifugal Filter Units (MWCO = 30 kDa). The molecular weight for the purified LF_N constructs confirmed by LC-MS.

Fluorophore Ligations.

scFv-AF647. The AF647 fluorophore was conjugated to the anti-XCR1 scFv by enzymatic ligation. The reaction was performed according to previously-developed conditions using SrtA* and peptide **1a** (*6*). The scFv-LPSTGG-H6 (μM) was combined with peptide **1a**, SrtA* (10 μM), and 0.5 M $CaCl_2$ (for a final concentration of 10 mM $CaCl_2$). The reaction was gently rotated for 45 min., followed by adding triple-rinsed Ni NTA agarose beads (50 μL per 1 mg of protein). The bead mixture was incubated for 15 min., followed by centrifugation (30 sec × 16,000 rpm) to collect the reaction supernatant. The beads were rinsed with successive rounds of PBS at pH 8.5 (3 × 0.5 mL). The rinses were combined and pushed through a 0.2 um syringe filter. To the mixture was added aqueous EDTA (0.5 M, 100 μL) for sequestering the $CaCl_2$. The reaction mixture was loaded onto HiLoad 16/600 Superdex 200 pg size exclusion column (GE), then eluted with PBS (Corning). Purified fractions of the scFv-AF647 constructs were analyzed by SDS-PAGE at 165 V for 36 min on an Invitrogen Bolt™ 4–12% Bis-Tris Plus Gel with Bolt™ MES SDS Running Buffer (1x). Gels were visualized by SimplyBlue™ SafeStain (Coomassie). Clean fractions were pooled and concentrated with Amicon® Ultra-15 Centrifugal Filter Units (MWCO = 15 kDa). The molecular weight of the purified LF_N constructs confirmed by LC-MS.

mPAC-AF647. The AF647 fluorophore was conjugated to the mPAC thiol using maleimide conjugation. AZDye™ 647 Maleimide (Click Chemistry Tools, 5 M, DMSO) was added to a solution of mPAC (100 µM) in PBS (Corning, pH 8.5). The reaction was gently rotated for 45 min., followed by filtering through a 0.2 um centrifugal filters (VWR), loading onto HiLoad 16/600 Superdex 200 pg size exclusion column (GE), and eluting with PBS (Corning, pH 7.5). Purified fractions of the mPAC-AF647 constructs were collected and analyzed by SDS-PAGE at 165 V for 36 min on an Invitrogen Bolt™ 4–12% Bis-Tris Plus Gel with Bolt™ MES SDS Running Buffer (1x). Gels were visualized by SimplyBlue™ SafeStain (Coomassie). Clean fractions were pooled and concentrated with Amicon® Ultra-50 Centrifugal Filter Units (MWCO = 50 kDa). The molecular weight of the purified mPAC-AF647 was confirmed by LC-MS.

scFv-mPAC-AF647. The AF647 fluorophore was conjugated to scFv-mPAC amine side chains using N-Hydroxysuccinimide (NHS). AZDye 647 NHS Ester (Click Chemistry Tools, 4 M, DMSO) was added to a solution of scFv-mPAC (10 µM) in PBS (Corning, pH 8.5). The reaction was gently rotated for 45 min., followed by filtering through a 0.2 um centrifugal filters (VWR), loading onto HiLoad 16/600 Superdex 200 pg size exclusion column (GE), and eluting with PBS (Corning, pH 7.5). Purified fractions of the scFv-mPAC-AF647 constructs were collected and analyzed by SDS-PAGE at 165 V for 36 min on an Invitrogen Bolt™ 4–12% Bis-Tris Plus Gel with Bolt™ MES SDS Running Buffer (1x). Gels were visualized by SimplyBlue™ SafeStain (Coomassie). Clean fractions were pooled and concentrated with Amicon® Ultra-50 Centrifugal Filter Units (MWCO = 50 kDa). The molecular weight of the purified scFv-mPAC-AF647 was confirmed by LC-MS.

Endotoxin testing and removal. Protein endotoxin levels were measured at 0.045 µg/mL using single-use cartridges (0.05 EU/mL, PTS2005) and the Endosafe® nexgen-PTS™ reader (Charles river). If endotoxin levels were > 0.015 EU/µg, previously-established procedures were used to remove endotoxin (*5*). In particular, treatment with Pierce™ High-Capacity Endotoxin Removal Resin reduced endotoxin levels with high protein recovery (~90%), which was repeated (1–3x) until sufficient endotoxin levels were reached (≤ 0.015 EU/µg).

Cell experiments. Wild-type CHO-K1 cells (ATCC CCL-61), XCR1-positive CHO cells (Creative Bioarray ACC-RG1782), and DC2.4 (Millipore, SCC142) cells were maintained in RPMI medium supplemented with 10% (v/v) FBS (ThermoFisher Scientific, MA, USA), 1% (v/v) pen/strep. B16-F10 murine melanoma cells (ATCC CRL-6475) were subculture as indicated by the provider, using Dulbecco's modified eagle's medium (DMEM, KATCC, VA, USA) completed with 10% FBS (ThermoFisher Scientific, MA, USA) + antibiotic-antimycotic cocktail (ThermoFisher Scientific, MA, USA), and incubated at 37 °C, 95% air, 5% CO_2 in humidified chamber. For *in vivo* tumor induction, the cells were passaged the day before to ensure they were in exponential growth phase.

Cell viability analysis. Cells were maintained at 37 ºC and 5% CO_2. CHO-K1 (ATCC) cells were maintained in F-12K media supplemented with 10% (v/v) fetal bovine serum and 1% penicillin/streptomycin. XCR1-CHO (Creative Bioarray) were maintained in RPMI-1640 media supplemented with 10% (v/v) fetal bovine serum and 10 µg/mL puromycin. Prior to an experiment, cells were plated at 1×10^3 cells/well in a 96-well flat-bottom plate and allowed to adhere overnight. Protein treatments were prepared in the corresponding cell culture medium, which included PA, scFv-mPAC, or scFv-mPAC[F427A] and serial dilutions of LF_N-DTA. The cells were resuspended with the protein treatments, followed by incubation for 72 h at 37 ºC and 5% CO_2. Cell viability was measured by the CellTiter-Glo luminescent assay (Promega) according to the manufacturer's procedure. Relative viability was inferred from the luminescence values. All counts were normalized to untreated cells and all experiments were done in triplicate.

Flow cytometry. Antibody staining was performed at a dilution of 1:100 for 25 minutes at 4 ºC in the presence of mouse Fc block (TruStain FcX™, anti-mouse CD16/32, BioLegend) in PBS containing 5% FBS. Individual cell populations were analyzed with antibodies purchased from BioLegend against: XCR1 (ZET), isotype (MPC-11), IFN-γ (MP6-XT22), TNF-α (XMG1.2), CD3 (17A2), B220 (RA3-6B2), CD11b (M1/70), CD11c (N418), F4/80 (BM8) and CD8a (53-6.7). Viability was assessed by LIVE/DEAD Fixable Aqua (Life Technologies). Cells were analyzed using a BD LSR Fortessa flow cytometer. Data were processed in FlowJo v10 and analyzed in GraphPad Prism v9.

Animal studies. All experiments were performed under an institute-approved IACUC protocol following federal, state, and local guidelines for the care and use of animals. C57BL6/J mice were procured from The Jackson Laboratory. 6- to 12-week-old female mice were used for these studies and were housed with free access to food and water *ad libitum* over the experiment. For the subcutaneous (s.c.) injections, the animals were shaved at the right flank and the tail base. In vivo NIRF imaging, shaving, injections, and tumor measurements were performed under anesthesia using 2-3% isoflurane inhalation along with O_2. The animals were sacrificed by CO_2 inhalation. In vivo NIRF imaging was performed using the In Vivo Imaging System IVIS (Perkin Elmer, USA). All compounds injected in mice were sterile or filtered using 0.22 µm.

Biodistribution. Concentrations of the AF647-labelled proteins were calculated using A280 and A650. The proteins (1 nmol) were subcutaneously administered to mice, in which half of the dose was given on each side of the tail base. The mice were evaluated for biodistribution to individual organs. At the indicated time points, whole organs were excised and imaged by IVIS fluorescence imaging (Caliper Life Sciences; excitation, 640 nm; emission, 680 nm). Images were processed using the Living Image software to determine radiant efficiency $((p\times s^{-1}\times cm^{-2}\times sr^{-1})/(\mu W\times cm^{-2}))$. For duplicate organs (i.e., spleen, lymph nodes, and kidney), the fluorescence signal was reported as the average mean fluorescence (± s.d.) of the two organs. Individual cell populations were further evaluated by flow cytometry, using spleens mashed through 70-µm filters (Corning), lysed in ACK buffer, and added to a 96-well U-bottom plate at 1×10^5 cells/well. The flow panel included the following fluorophores: protein analytes (AF647), viability (LIVE/DEAD™ Fixable Aqua), CD3 (BV510), B220 (BV510), CD11b (PE), CD11c (BV421), F4/80 (PE-Cy7) and CD8a (BV711).

ELISpot. On days 0 and 14, mice (C57Bl/6, n = 5, F, 5–7 weeks) were primed and boosted with s.c. injections, in which half of the dose was given on each side of the tail base. The treatments comprised proteins (6 pmol, PA or scFv-mPAC; 30 pmol, LF_N-$OVA_{252-270}$) mixed with 25 μg c-di-GMP (Invivogen). On day 21, antigen-specific immune responses were analyzed by IFN-γ ELISpot (BD Biosciences) according to the manufacturer's instructions. On the final day of the experiment, spleens from vaccinated animals were excised, mashed through 70-μm filters (Corning), lysed in ACK buffer, and plated in a pre-coated 96-well ELISPOT plate at 5×10^6 splenocytes/well (i.e., one spleen per well). CD8 T cells were stimulated with the corresponding epitope peptide (Table S3, $OVA_{257–264}$) at 10 μg/mL in complete RPMI-1640 medium (10% FBS + 1% penicillin/streptomycin). The plate was incubated for 24 h at 37 °C and 5% CO_2, then spot-forming units (s.f.u.) were imaged and counted. Data from two independent experiments of 5 animals/group were pooled to reach full cohort size (n = 10).

Intracellular cytokine staining. Mice (C57Bl/6, 5–7 weeks) were vaccinated with s.c. injections, in which half of the dose given on each side of the tail base. Peripheral blood was analyzed 7 d after each boost by intracellular cytokine staining of IFN-γ and TNF-α. Peripheral blood was collected, red blood cells were lysed, and lymphocytes were plated with 10 μg/mL of the epitope peptide (Table S3, $OVA_{257–264}$). After 2 h incubation at 37 °C and 5% CO2, brefeldin A was added and samples were returned to incubation for an additional 4 h. Samples were stained with a fixable viability dye followed by extracellular staining. Using a fixation/permeabilization kit (BD Biosciences), samples were prepared for intracellular antibody staining and assessed by flow cytometry.

MHC tetramer staining. Mice (C57Bl/6, 5–7 weeks) were vaccinated with s.c. injections, in which half of the dose given on each side of the tail base. Peripheral blood was analyzed 7 d after each boost by MHC tetramer staining to detect antigen-specific $CD8^+$ T cells. Peripheral blood was collected, red blood cells were lysed, and lymphocytes were plated with H-2Kb/SIINFEKL tetramer stain and 50 nM dasatinib. After 30 min incubation at 37 °C and 5% CO2, samples were prepared for extracellular antibody staining and assessed by flow cytometry.

Tumor therapy. Thirty female C57Bl/6J mice, 5–7 weeks-old, 18-20 g were s.c injected in the flank with 3×10^5 B16-F10 cells suspended in 100 μL of complete medium. At Day 4 post tumor induction, the animals were sorted into groups (n = 8–10 per group (Fig. A). At Day 4, 10, 16 and 22 the mice were s.c vaccinated at both sides of the tail base with 2 × 25 μL of c-di-GMP (25 μg) combined with either Trp1 + gp100 (50 pmol each) or LF_N-Trp1-gp100 (50 pmol) + scFv mPAC (10 pmol), then intraperitoneally (i.p) injected with 200 μg (100μL) of anti-PD1 CD279 antibody (BioXCell, NH, USA). Animals in the naive group were i.p injected with anti-PD1 and s.c. injected with saline solution. Animals were weighed every 2 days and sacrificed if they lost ≥ 20% of their weight. To determine the survival, the tumor surface (L × W) was measured daily using a caliper, and the animals were then sacrificed when the tumor reached 100 mm^2.

Statistical analysis. Calculations were made using GraphPad Prism 8. For splenocytes quantifications, comparisons were done using one-way ANOVA with Fisher's least significance difference test. For *in vivo* tumor growth monitoring, calculations were performed both using a two-way ANOVA with Dunnett's multiple comparison versus the naive group (until Day 16 for Day-to-Day comparisons) or two-way ANOVA with Tuckey comparison (from Day 0 to the end for curve comparisons) (*p< 0.05; **p< 0.005; ***p<0.001 and ****p< 0.0001). Kaplan-Meier percent survival curves were compared using the log-rank Mantel-Cox test.

IV. REFERENCES

1. N. L. Truex *et al.*, Automated Flow Synthesis of Tumor Neoantigen Peptides for Personalized Immunotherapy. *Sci. Rep.* **10**, 723 (2020).

2. A. J. Mijalis *et al.*, A Fully Automated Flow-Based Approach for Accelerated Peptide Synthesis. *Nat. Chem. Biol.* **13**, 464–466 (2017).

3. N. Hartrampf *et al.*, Synthesis of Proteins by Automated Flow Chemistry. *Science* **368**, 980–987 (2020).

4. C. J. Miller, J. L. Elliott, R. J. Collier, Anthrax protective antigen: prepore-to-pore conversion. *Biochemistry* **38**, 10432–10441 (1999).

5. Z. Lu *et al.*, IgG-Engineered Protective Antigen for Cytosolic Delivery of Proteins into Cancer Cells. *ACS Cent. Sci.* **7**, 365–378 (2021).

6. J. J. Ling, R. L. Policarpo, A. E. Rabideau, X. Liao, B. L. Pentelute, Protein Thioester Synthesis Enabled by Sortase. *J. Am. Chem. Soc.* **134**, 10749–10752 (2012).
